# Optimal exercise prescription parameters in Schroth training for adolescent idiopathic scoliosis: a systematic review and meta-analysis

**DOI:** 10.3389/fsurg.2026.1796813

**Published:** 2026-04-22

**Authors:** Daoquan Guan, Yahui Liu, Tongwu Yu, Wen Cheng

**Affiliations:** 1 Anhui Communications Vocational & Technical College, Hefei, China; 2 Kyungwoon University, Gumi-si, Republic of Korea; 3 Capital University of Physical Education and Sports, Beijing, China; 4 Shinhan University, Uijeongbu-si, Gyeonggi-do, Republic of Korea

**Keywords:** adolescent idiopathic scoliosis, Cobb angle, dose response, meta-analysi, quality of life, Schroth exercises, systematic review

## Abstract

**Background:**

Adolescent idiopathic scoliosis (AIS) affects 0.5%–5% of the adolescent population, representing the most common spinal deformity in this age group. This systematic review and meta-analysis aimed to determine optimal exercise prescription parameters for Schroth training in AIS by examining dose-response relationships between exercise frequency, duration, and clinical outcomes.

**Methods:**

Systematic searches of PubMed, Scopus, Web of Science, and SPORTDiscus were conducted following PRISMA 2020 guidelines. Randomized controlled trials examining Schroth interventions in adolescents aged 10–18 years with idiopathic scoliosis were included. Network meta-analysis was performed using MetaInsight platform for Cobb angle outcomes, with traditional pairwise meta-analyses conducted for all outcomes using standardized mean differences with 95% confidence intervals.

**Results:**

15 randomized controlled trials encompassing 620 participants were included. The overall meta-analysis demonstrated Schroth exercises produced statistically significant Cobb angle improvements (SMD = −0.52, *p* < 0.0001; I^2^ = 0%). Subgroup analysis revealed dose-response relationships favouring moderate exercise frequencies (3–4 sessions/week), which showed the largest pooled effect (SMD = −0.58, I^2^ = 3%). Duration analysis demonstrated medium duration interventions (46–75 min) provided the most precise improvements (MD = −2.92°), while optimal frequency was the moderate (MD = −2.79°, 95% CI: −4.05, −1.48). Combined subgroup analysis identified moderate frequency plus medium duration as the most robust combination (SMD = −0.65, I^2^ = 10%). Health-related quality of life outcomes showed non-significant improvements with substantial heterogeneity (SMD = 0.52, *p* = 0.43; I^2^ = 93%). Secondary outcomes showed statistically significant improvements in trunk rotation (SMD = −0.86, *p* = 0.002; I^2^ = 22%) and cosmetic appearance perception (SMD = −0.73, *p* = 0.01; I^2^ = 0%), while postural stability measures showed non-significant effects (SMD = 0.08; *p* = 0.81; I^2^ = 68%). Publication bias assessment using Egger's test shows no statistically significant funnel plot asymmetry (*p* = 0.745).

**Conclusions:**

Exploratory subgroup and network meta-analyses suggest that moderate frequency Schroth exercises (3–4 sessions/week) combined with medium duration sessions (46–75 min) may represent optimal parameters for Cobb angle improvement in AIS. Non-linear dose-response patterns show diminishing returns at higher frequencies, challenging conventional exercise prescription assumptions. These findings require validation through prospective studies with pre-specified dose-stratification examining long-term effectiveness, cost-effectiveness, and patient adherence across diverse healthcare contexts before broad clinical implementation.

## 1 Introduction

Adolescent idiopathic scoliosis (AIS) represents the most common structural spinal deformity affecting the paediatric population. AIS is characterized by a three-dimensional deviation of the spinal axis involving lateral curvature, vertebral rotation, and sagittal plane alterations (1–3). This complex spinal condition manifests as a laterally rotated curvature of the spine with a Cobb angle of ≥10° in the coronal plane, becoming apparent in generally healthy children during periods of rapid growth around puberty (2, 4). The prevalence of AIS varies globally, with epidemiological studies indicating rates between 0.5% and 5% in the general adolescent population (4, 5). AIS demonstrates marked female predominance that increases substantially with curve severity, ranging from a 1.4:1 female-to-male ratio in mild curves (10°–20°) to 10:1 in curves exceeding 30° (4, 5). Recent surveys in mainland China have documented a prevalence of 1.23% among primary and secondary school students, with the condition emerging as the third most prevalent health issue among adolescents, following obesity and myopia (3).

The aetiology of AIS remains multifactorial and poorly understood, with research suggesting a complex interplay of genetic predisposition, spinal biomechanics, neurological factors, hormonal regulation, biochemical influences, and environmental factors (4, 5). Various theories have been proposed to explain the development and progression of AIS, including the melatonin hypothesis, genetic susceptibility, and environmental influences, though no single theory fully explains the onset and progression of this condition (4). The natural history of AIS demonstrates significant variability, with prospective studies indicating that 68% of adolescent idiopathic curvatures progress beyond skeletal maturity, particularly thoracic curves greater than 50° which progress at an average rate of 1° per year (1). While traditionally described as a pain-free condition, contemporary research has challenged this notion, with studies demonstrating that approximately 23% of patients with AIS present with back pain at initial diagnosis, and an additional 9% develop pain during the course of their condition (1, 2).

The clinical presentation of AIS extends beyond structural deformity to encompass significant psychosocial implications, including restricted social life, increased psychiatric consultations, higher rates of eating disorders, and compromised quality of life. Patients typically with visible postural asymmetries including uneven shoulder levels, waist asymmetry, rib prominence, and truncal shift, which can result in loss of self-confidence and depression (1, 3, 4). In severe cases, individuals may experience respiratory difficulties, cardiovascular compromise, and progressive disability, particularly when curves exceed 50° and progress into adulthood (3, 4). The magnitude of spinal curvature is classified using the Cobb angle measurement, with curves up to 25° considered mild, 25°–45° classified as moderate, and curves above 45° categorized as severe scoliosis (1, 2).

Treatment approaches for AIS are stratified based on curve severity, skeletal maturity, and progression risk, encompassing observation, conservative management, and surgical intervention (4, 6). The International Society on Scoliosis Orthopaedic and Rehabilitation Treatment (SOSORT) guidelines advocate for conservative management combining bracing with physiotherapeutic scoliosis-specific exercises (PSSE) for patients with Cobb angles between 10° and 45°, reserving surgical intervention for severe progressive curves (3, 7). SOSORT has formally recognized PSSE as an evidence-based therapeutic intervention, with exercise therapy gaining significant recognition as a cost-effective treatment modality (3, 7, 8) that offers numerous advantages over prolonged bracing, including improved patient compliance, enhanced aesthetic perception, reduced psychological burden, and overall improvement in quality of life.

Among the various PSSE approaches, the Schroth method has emerged as the most extensively studied and widely implemented therapeutic intervention internationally (7, 9–11). Originally developed by Katharina Schroth in 1921, this three-dimensional exercise approach integrates postural correction, sensorimotor retraining, and corrective breathing patterns to restore spinal alignment and stability through targeted muscular contractions and specific breathing techniques (7, 11). The Schroth method employs a personalized approach utilizing breathing and self-correcting techniques performed in front of mirrors, supplemented with additional equipment such as wall poles, sticks, and Swiss balls, with the primary goal of eliminating muscle imbalances by strengthening and stretching soft tissues while directing inhaled air toward the concave side of the ribcage to facilitate thoracic expansion and derotation.

Contemporary evidence demonstrates that Schroth exercises produce significant improvements in multiple clinical outcomes, including Cobb angle reduction, enhanced quality of life measures, and improved trunk rotation compared to standard care or observation alone (2, 3). Meta-analytic evidence has shown that Schroth exercises significantly decrease the Cobb angle (SMD = −3.32°, 95% CI: −4.15, −2.50; *p* < 0.001) and improve quality of life scores (SMD = 2.80, 95% CI [1.53, 4.06]; *p* < 0.001) when compared to conventional physical therapy (3).

Despite the growing evidence base supporting Schroth exercise effectiveness, substantial heterogeneity exists in exercise prescription parameters across existing studies, creating significant uncertainty for clinicians regarding optimal treatment protocols (2, 5, 9, 12, 13). Current literature reveals considerable variation in training frequency (ranging from 1 to 7 sessions per week), session duration (15–90 min), total intervention duration (4–52 weeks), and progression protocols, with limited guidance on the minimum effective dose or optimal parameter combinations (3, 12–14). This variability in exercise prescription potentially limits treatment effectiveness, compromises patient outcomes, and hinders the development of standardized clinical protocols.

The absence of systematic evaluation of exercise prescription parameters represents a critical knowledge gap in the current literature, as understanding the dose-response relationships between exercise frequency, intensity, duration, and progression protocols is essential for developing evidence-based treatment guidelines. Furthermore, while individual studies have demonstrated the efficacy of various Schroth exercise protocols, the relative effectiveness of different parameter combinations and the identification of optimal prescription thresholds remain unexplored. This systematic review and meta-analysis aims to address this critical knowledge gap by providing the first comprehensive evaluation of exercise prescription parameters in Schroth training for adolescents with idiopathic scoliosis. By synthesizing existing evidence and examining the relationships between exercise prescription variables and clinical outcomes, this review will establish evidence-based recommendations for optimal Schroth exercise protocols, ultimately improving treatment efficiency, enhancing patient adherence, and supporting the development of cost-effective healthcare delivery protocols for this prevalent adolescent condition.

## 2 Methods

### 2.1 Protocol registration and reporting

**Figure 1:**
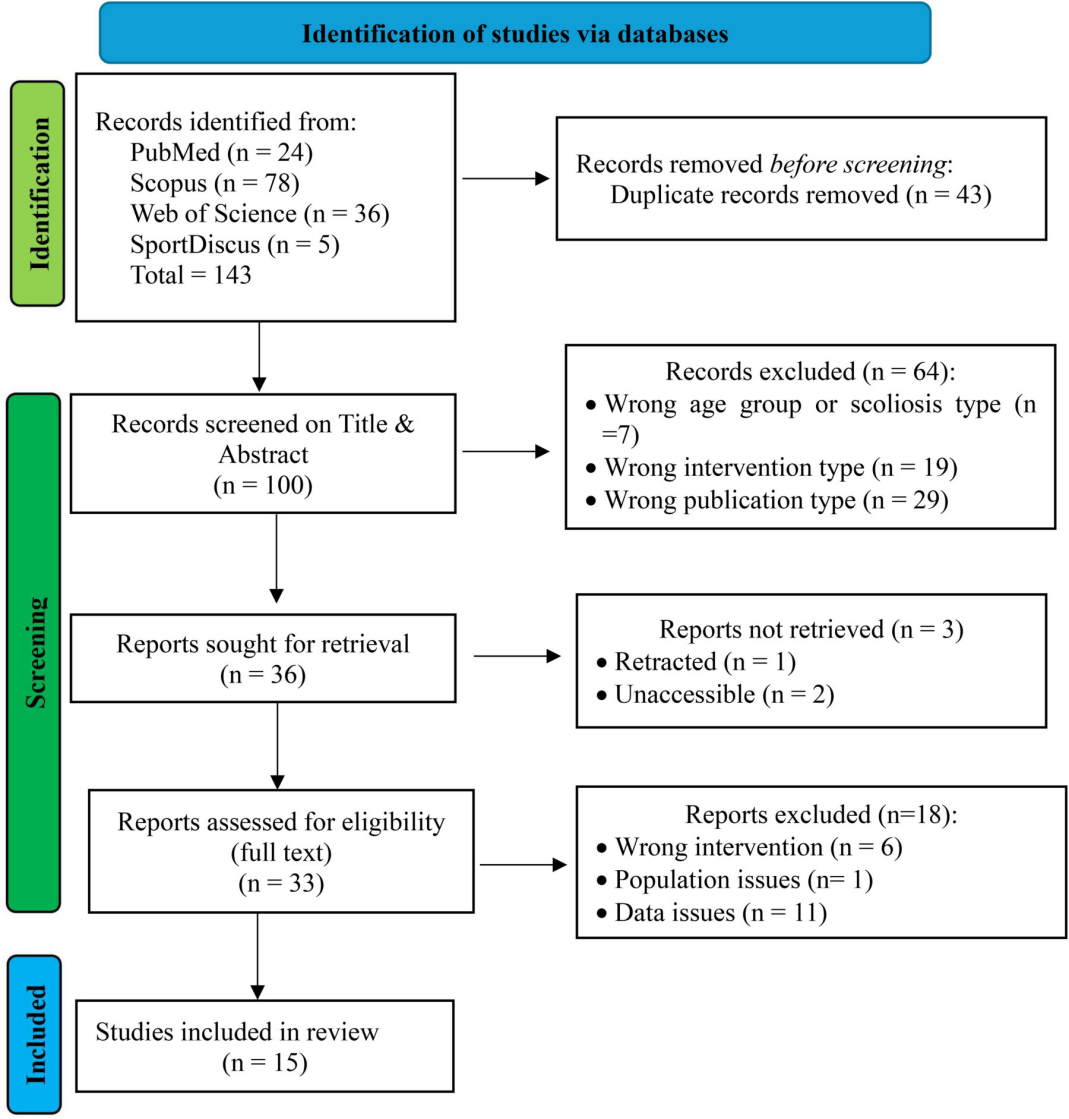
PRISMA 2020 flow diagram for the systematic review.

This systematic review protocol was prospectively registered with the International Platform of Registered Systematic Review and Meta-analysis Protocols (INPLASY), under registration number INPLASY202570022 (doi: 10.37766/inplasy2025.7.0022). The protocol is publicly accessible at https://inplasy.com/inplasy-2025-7-0022/. This systematic review adhered to the Preferred Reporting Items for Systematic Reviews and Meta-Analyses (PRISMA) 2020 guidelines for search strategy, study selection, data extraction, and result reporting (Figure 1).

### 2.2 Eligibility criteria

The eligibility criteria for study inclusion were established *a priori* based on the PICOS framework to ensure systematic identification of relevant randomized controlled trials examining Schroth exercise interventions in AIS (15–17). Both inclusion and exclusion criteria were applied consistently during the study selection process to maintain methodological rigor.

#### 2.2.1 Inclusion criteria

Studies were included if they were randomized controlled trials (parallel-group or crossover designs) conducted in any setting (single/multi-centre, clinical/outpatient/home-based). The target population comprised adolescents aged 10–18 years with confirmed idiopathic scoliosis (Cobb angle ≥10°), any curve pattern (thoracic, lumbar, thoracolumbar, double major), any skeletal maturity level (Risser 0–5), and both sexes.

Interventions must be Schroth exercise training based on recognized Schroth principles (three-dimensional postural correction, breathing techniques, curve-specific protocols) with documented exercise prescription parameters (frequency, duration, or intensity) and minimum four-week duration (3, 9, 12). Eligible comparators included standard care/observation, different Schroth prescriptions, other conservative interventions (bracing, alternative exercises), or waitlist controls, provided they matched the intervention group in population characteristics, outcome timepoints, and intervention duration.

Studies must report at least one primary outcome (Cobb angle, quality of life, or trunk rotation), be published in peer-reviewed English-language journals (January 2014–June 2025), and provide sufficient statistical data for meta-analysis (means, standard deviations, or extractable effect sizes) with minimum ten participants per group and adequate randomization procedures.

#### 2.2.2 Exclusion criteria

Studies were excluded if they: (1) included adult populations (>18 years), non-idiopathic scoliosis (congenital, neuromuscular, degenerative), or post-surgical patients; (2) examined non-Schroth interventions, passive treatments, or combined interventions without separate Schroth analysis; (3) lacked appropriate control groups or reported outcomes of interest; (4) had intervention durations <4 weeks; or (5) were duplicate publications or had inadequate sample sizes compromising methodological rigor.

### 2.3 Search strategy

A comprehensive search strategy was implemented across four electronic databases (PubMed/MEDLINE, Web of Science Core Collection, Scopus, and SPORTDiscus) to identify relevant randomized controlled trials examining Schroth exercise interventions for AIS, with publication dates from January 1, 2014, to June 30, 2025 (17). The search strategy combined three core concept categories using Boolean operators. Population terms included “adolescent idiopathic scoliosis,” “AIS,” “juvenile idiopathic scoliosis,” and “scoliosis,” linked with OR operators. Intervention terms encompassed “Schroth,” “Schroth exercise,” “Schroth method,” “Schroth therapy,” “physiotherapeutic scoliosis-specific exercise,” and “PSSE,” also combined with OR operators. Study design terms comprised “randomized controlled trial,” “randomized,” “controlled trial,” “clinical trial,” and “RCT,” similarly linked with OR operators. These three concept groups were combined using AND operators to create the final search string. The search strategy was adapted for each database using database-specific subject headings, such as Medical Subject Headings (MeSH) terms and appropriate search operators (Title, Title and Abstract, abstract, etc) while maintaining consistency in core concepts to optimize retrieval sensitivity and precision. Complete search strategies for each database, including specific search strings, filters applied, and results obtained, are detailed in Appendix 1.

### 2.4 Study selection process

The study selection process was conducted systematically in two sequential stages using EPPI-Reviewer software (Version: 6.17.0.0 - https://eppi.ioe.ac.uk/eppireviewer-web/home) (18, 19), with independent screening performed by two reviewers to ensure objectivity and minimize selection bias. Disagreements between reviewers were resolved through structured discussion based on the appropriateness of studies according to the inclusion and exclusion criteria (17, 18, 20). During the initial title and abstract screening phase, a structured 8-category coding framework was employed to systematically evaluate study eligibility. Studies were classified as either meeting inclusion criteria, requiring full-text assessment due to insufficient information, or excluded based on predetermined criteria (EXCLUDE on Wrong Age Group or Scoliosis Type; Wrong Intervention Type; Wrong Study Design; No Relevant Outcomes; Insufficient Intervention Duration; Wrong Publication Type). A deliberately inclusive approach was adopted at this preliminary stage, with studies of uncertain relevance advanced to full-text review to minimize the risk of inadvertently excluding potentially eligible research. The comprehensive full-text screening stage involved detailed assessment of studies against the complete eligibility criteria using a refined 8-item evaluation framework (EXCLUDE on Population issues; Wrong intervention; Inappropriate study design; Missing primary outcomes; Insufficient exercise prescription data; Inadequate data for meta-analysis; Duplicate/poor quality; INCLUDE on full study). Only studies demonstrating clear adherence to all inclusion criteria and providing sufficient methodological detail and statistical data for quantitative synthesis were retained for final inclusion. Throughout the selection process, detailed documentation was maintained regarding screening decisions and rationales.

### 2.5 Data extraction

Standardized data extraction forms were developed and piloted based on Cochrane Handbook guidelines and established recommendations for exercise prescription systematic reviews (16, 17, 21–23). Two independent reviewers extracted data from all included studies using a comprehensive extraction framework designed to capture exercise prescription parameters according to the FITT-VP (Frequency, Intensity, Time, Type, Volume, and Progression) model, essential for conducting meaningful dose-response analyses (3, 9, 17). All extracted data was cross-verified between reviewers, with discrepancies resolved through structured discussion.

Table 1 summarizes the comprehensive data extraction categories employed in this review.

**Table 1:** Data extraction categories and variables.

Category		Extracted variables
Study identification		Primary author, publication year, country of origin, study design characteristics, sample sizes for all study arms
Population characteristics		Age (mean ± SD), sex distribution, baseline Cobb angle (primary and secondary curves), curve severity classification (mild 10–25°, moderate 26-40°, severe ≥41°), curve pattern (thoracic, lumbar, thoracolumbar, double major), skeletal maturity (Risser grade), previous treatment history (bracing, prior exercises)
Exercise prescription	Frequency	Supervised sessions per week, total sessions over intervention period, session scheduling pattern
	Intensity	Exercise difficulty level, progression rate, rating of perceived exertion scales, resistance levels
	Duration	Session length (minutes), active exercise time within sessions, total intervention duration (weeks), rest periods between exercises
	Type	Specific Schroth method variant, main exercise categories, breathing component duration, postural correction elements
	Volume	Total exercise volume (sessions × duration × weeks), weekly exercise load, cumulative exposure time
	Progression	Progression criteria, methods of advancement, individualization approaches
Supervision and context		Supervision level, home exercise components (documented as contextual information), sports participation, additional physical activities
Comparator characteristics		Control intervention type, co-interventions permitted, attention control measures, intervention duration matching, measurement timepoint matching
Primary outcomes		Cobb angle measurements (baseline, post-intervention values); Quality of life scores using validated instruments (SRS-22, SRS-23, SAQ) at all timepoints (24).
Secondary outcomes		Trunk rotation (assessment method: scoliometer, surface topography), balance and postural stability measures, postural parameters and asymmetry indices, pulmonary function (forced vital capacity), pain scores (validated scales), cosmetic appearance perception measures; all with documented measurement timepoints

SD, standard deviation; SRS-22, scoliosis research society-22 questionnaire; SRS-23, scoliosis research society-23 questionnaire; SAQ, spinal appearance questionnaire.

Supervised session parameters were prioritized for primary dose-response analyses as these were most consistently reported across studies and represent the core prescription decision facing clinicians regarding professional resource allocation. Home exercise components (typically 10–15 min daily of self-directed exercises in some protocols) were documented as contextual information when reported. Sports participation and additional physical activities were extracted when available to contextualize total physical activity load.

Data were extracted in original units without conversion to maintain precision and enable appropriate statistical pooling (17, 25, 26). Data extraction forms underwent quality checks including verification of calculations and identification of inconsistencies or missing information requiring clarification from study authors. The extraction process maintained an audit trail documenting all decisions and modifications to support transparency and reproducibility.

### 2.6 Statistical Analysis

#### 2.6.1 Data synthesis and meta-analysis approach

All statistical analyses were conducted using multiple complementary platforms to ensure comprehensive evaluation: R software (version 4.3.0) for data cleaning and preparation, Review Manager Web (RevMan Web) for traditional meta-analyses (16, 17). Random-effects meta-analysis using the Restricted Maximum-Likelihood method was employed as the primary analytical approach, accounting for expected heterogeneity between studies due to varying exercise prescription parameters, population characteristics, and methodological differences across included trials (27–30).

#### 2.6.2 Effect size calculation and outcome measures

For continuous outcomes, standardized mean differences (SMD) using Hedges’ g were calculated to account for different measurement scales across studies, with 95% confidence intervals for all effect estimates. Effect sizes were interpreted using established thresholds: small (0.2), moderate (0.5), and large (0.8) effects for SMD values (17, 31).

#### 2.6.3 Network meta-analysis

Network meta-analysis was conducted exclusively for Cobb angle outcomes due to sufficient data availability (*n* = 13 studies) using MetaInsight platform, which employs mean differences as the default effect measure (20, 32, 33). Out of the 15 studies included, 13 reported Cobb angles results. The Bayesian approach was selected for computational efficiency and interpretability (5, 34). Dose category thresholds were defined based on the distribution of prescription parameters observed across included studies and alignment with clinical practice conventions: frequency categories reflect clinically distinct supervision intensities (low: 1–2 sessions representing minimal contact; moderate: 3–4 reflecting standard outpatient practice; high: ≥5 representing near-daily protocols), while duration categories reflect natural breakpoints in the data (short: ≤45 min; medium: 46–75 min; long: ≥76 min)."Treatment rankings were generated using surface under the cumulative ranking (SUCRA) probabilities (35, 36). Network connectivity and coherence were not assessed because there were no closed loops required for node-splitting approaches (20, 32, 33).

#### 2.6.4 Traditional pairwise meta-analysis and subgroup analysis

Traditional pairwise meta-analyses were conducted using RevMan Web for all outcome measures (17, 26). Subgroup analyses examined exercise frequency (low, moderate, high), session duration (short, medium, long), and combined prescription parameters to explore dose-response relationships (16, 17). Heterogeneity was assessed using I^2^ statistic: <25% (low), 25–50% (moderate), 50–75% (substantial), >75% (considerable). Tau-squared (*τ*^2^) measured between-study variance, with statistical significance evaluated using chi-squared test (*p* < 0.10) (37, 38). For all pairwise meta-analyses, post-intervention values were used as the primary data input, with change-from-baseline values used where post-intervention data were unavailable, consistent with Cochrane recommendations for continuous outcomes.

#### 2.6.5 Risk of bias assessment

**Figure 2:**
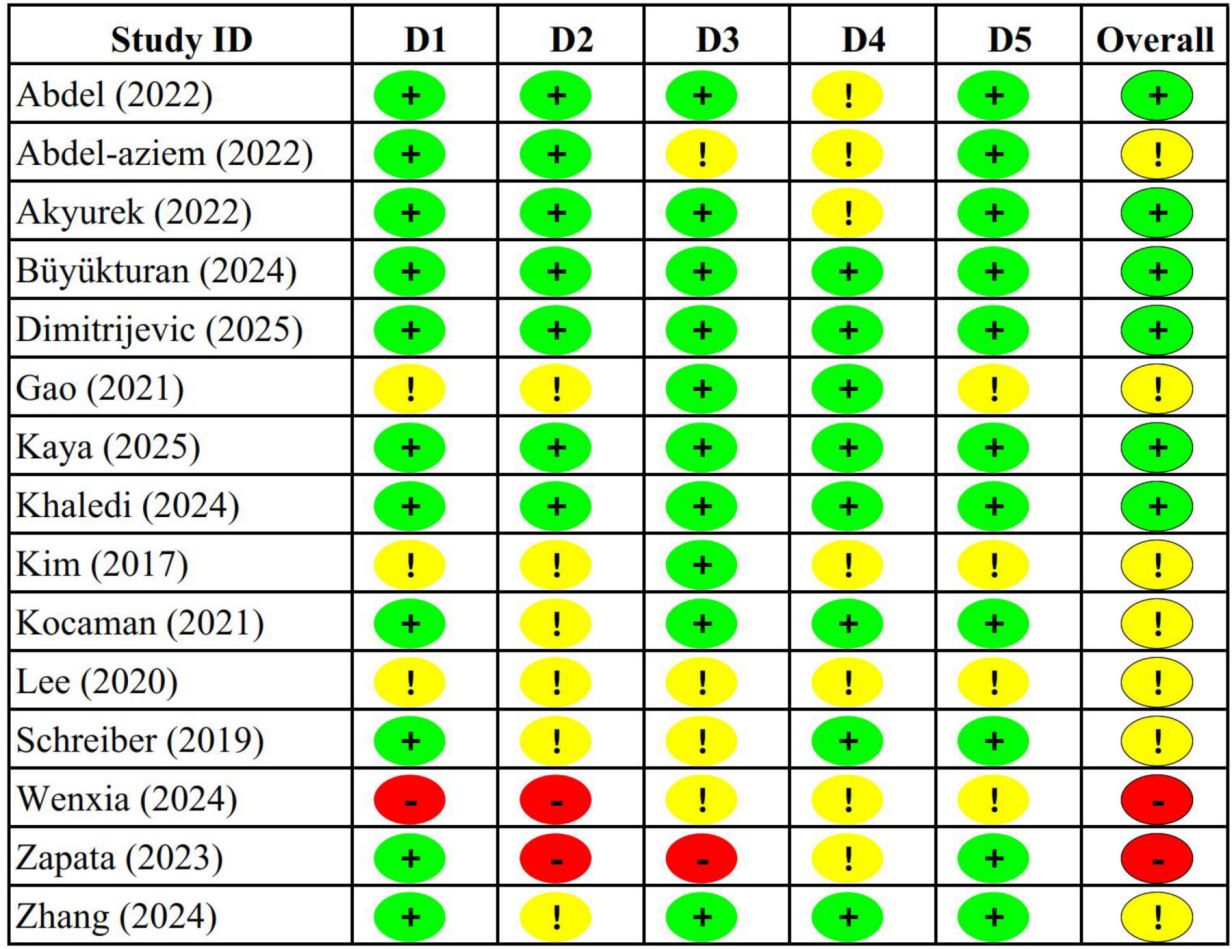
Risk of bias analysis of the studies. D1 = Randomisation process; D2 = Deviations from the intended interventions; D3 = Missing outcome data; D4 = Measurement of the outcome; D5 = Selection of the reported result; Red = High; Yellow = Some concerns; Greem = Low.

Methodological quality was assessed using the Cochrane Risk of Bias tool for randomized trials (RoB 2.0) (39–41). The five domains evaluated were: (1) randomization process, (2) deviations from intended interventions, (3) missing outcome data, (4) outcome measurement, and (5) selection of reported results, with judgements of “Low”, “High” risk, or “Some concerns”. Two reviewers independently assessed risk of bias for each study, with disagreements resolved through structured discussion (Figure 2).

#### 2.6.6 Publication bias assessment

Publication bias was assessed exclusively for Cobb angle outcomes (*n* = 13 studies), meeting the recommended threshold of ≥10 studies for reliable testing (42–44). Multiple complementary approaches were employed using R software with the meta and metafor packages: visual assessment through funnel plot examination, Egger's regression test as the primary statistical test for funnel plot asymmetry (41, 45) and trim-and-fill analysis to estimate potentially missing studies and provide adjusted effect size estimates. Publication bias test results were interpreted cautiously, recognizing that funnel plot asymmetry may reflect genuine heterogeneity, small-study effects, or methodological differences rather than publication bias alone.

## 3 Results

### 3.1 Study Selection

The comprehensive search strategy yielded 143 records across four databases, with Scopus contributing the largest proportion (*n* = 78), followed by Web of Science (*n* = 36), PubMed/MEDLINE (*n* = 24), and SPORTDiscus (*n* = 5) (see Appendix 1). Following systematic duplicate removal of 43 records, 100 unique records underwent title and abstract screening. The initial screening excluded 64 records, leaving 36 reports for full-text assessment. During full-text evaluation, 33 reports underwent detailed eligibility assessment, with 3 reports inaccessible despite contact attempts with authors and institutional libraries. Eighteen additional reports were excluded (6 for wrong intervention characteristics, 1 for population issues, 11 for data insufficiency), resulting in 15 studies (46–60) meeting all inclusion criteria for the systematic review and meta-analysis. This final selection represents a substantial evidence base examining Schroth exercise interventions in adolescent idiopathic scoliosis.

### 3.2 Study characteristics and population characteristics

The 15 included studies were randomized controlled trials encompassing a total of 620 participants with adolescent idiopathic scoliosis, representing a substantial international evidence base spanning multiple continents and healthcare systems. The studies were published between 2017 and 2025, with the majority published within the last five years, indicating growing research interest in Schroth exercise interventions for adolescent idiopathic scoliosis (see Table 2). Geographically, the included studies demonstrated global representation, with the largest contribution from Turkey (*n* = 4), followed by China (*n* = 3), South Korea and Saudi Arabia (*n* = 2), while the rest were single studies from Canada, United States, Serbia, and Iran, reflecting the international adoption and investigation of Schroth exercise protocols (see Table 2).

**Table 2:** Study identification and population characteristics.

Study ID	Country	Sample size	Age (years)	Sex distribution	Baseline cobb angle	Curve severity	Risser grade
Abdel et al. (46)	Saudi Arabia	INT=22, CON=23	INT=14.68 ± 1.72, CON=15.04 ± 1.66	INT = M/F(9/13), CON = M/F(8/15)	INT=15.59 ± 2.66, CON=16.32 ± 2.39	Mild (10–20°)	Risser 2–4
Abdel-aziem et al. (47)	Saudi Arabia	INT=27, CON=25	INT=14.74 ± 1.79, CON=15.04 ± 1.81	INT = M/F(8/19), CON = M/F(7/18)	INT=18.59 ± 2.66, CON=19.32 ± 2.69	Mild (10–20°)	NR
Akyurek et al. (48)	Turkey	INT=15, CON=14	INT=13.73 ± 1.83, CON=13.86 ± 1.86	All female	INT=18.87 ± 7.16, CON=20.00 ± 7.11	Mild (10–45°)	NR
Büyükturan et al. (49)	Turkey	INT=15, CON=16	INT=14.0 ± 1.9, CON=14.2 ± 2.0	INT = M/F(3/12), CON = M/F(4/12)	INT=22.8 ± 3.1, CON=20.4 ± 2.2	Mild-Moderate	INT=2.50 ± 1.65, CON=2.71 ± 2.05
Dimitrijevic et al. (50)	Serbia	INT=17, CON=17	INT=14.11 ± 1.02, CON=13.41 ± 1.63	INT = M/F(5/12), CON = M/F(5/12)	INT=30.18 ± 8.19, CON=30.24 ± 6.51	Moderate	INT=2 ± 1.28, CON=1.65 ± 1.45
Gao et al. (51)	China	Schroth=43, CON=21	Schroth=15.1 ± 1.6, Obs=15.8 ± 1.5	Schroth = M/F(7/36), Obs = M/F(4/17)	Schroth=28.9 ± 5.5, Obs=27.8 ± 4.1	Moderate	Schroth: 3 = 9, 4 = 19, 5 = 15; Obs: 3 = 5, 4 = 12, 5 = 4
Kaya et al. (52)	Turkey	Schroth=34, PNF=33	Schroth=13.8 ± 1.6, PNF=14.1 ± 1.8	Schroth = M/F(10/24), PNF = M/F(8/25)	Schroth=16.2 ± 2.7, PNF=17.2 ± 4.2	Mild	Schroth = NR, PNF = NR
Khaledi et al. (53)	Iran	SE + ASSE=15, SE = 15, CON=10	SE + ASSE=16.33 ± 0.90, SE = 16.27 ± 1.44, CON=15.4 ± 1.51	All males	SE + ASSE=16.45 ± 5.25, SE = 15.09 ± 4.41, CON=11.32 ± 0.96	Mild	0–3 included
Kim et al. (54)	South Korea	SERME=8, SE = 7	SERME=17.75 ± 4.71, SE = 15.57 ± 2.70	SERME = M/F(2/6), SE = M/F(3/4)	SERME=24.49 ± 8.32, SE = 27.16 ± 12.44	Mild	NR
Kocaman et al. (55)	Turkey	INT=14, CON=14	INT: 14.07 ± 2.37, CON: 14.21 ± 2.19	INT: M = 4 (28.6%), F = 10 (71.4%); CON: M = 3 (21.4%), F = 11 (78.6%)	Thoracic: INT: 17.64 ± 4.01, CON: 17.29 ± 3.45	Mild	INT: 1.64 ± 1.34, CON: 1.78 ± 1.19
Lee et al. (56)	South Korea	INT=8, CON=7	INT=18.88 ± 3.06, CON=24.14 ± 12.69	INT=1M/7F, CON=1M/6F	INT=22.11 ± 7.58, CON=22.17 ± 7.27	Mild to Moderate	NR
Schreiber et al. (57)	Canada	INT=25, CON=25	INT=13.5 ± 1.4, CON=13.3 ± 1.3	INT=2/23 (8/92), CON=1/24 (4/96)	INT=29.1 ± 8.9, CON=27.9 ± 8.8	Moderate	INT=1.76 ± 1.35, CON=1.44 ± 1.34
Wenxia et al. (58)	China	INT=17, CON=14	INT=12.53 ± 1.32, CON=13.43 ± 2.27	INT = M:F(6:11), CON = M:F(2:12)	INT=21.58 ± 8.15, CON=18.00 ± 5.42	Mild	INT: 12 with 0–2, 5 with >3; CON: 10 with 0–2, 4 with >3
Zapata et al. (59)	United States	INT=37, CON=37	INT: 12.7 ± 1.3, CON: 12.1 ± 1.0	INT: M/F (6/31), CON: M/F (5/32)	INT: 24 ± 4, CON: 25 ± 4	Mild	INT: NR, CON: NR
Zhang et al. (60)	China	INT=31, CON=29	INT: 13.42 ± 1.06, CON: 13.97 ± 1.21	INT: M/F (7/24), CON: M/F (5/24)	INT: 19.90 ± 2.26, CON: 20.21 ± 1.76	Mild	INT: NR, CON: NR

INT, intervention group; CON, control group; OBS, observation group; SE, Schroth exercise; ASSE, e asymmetric spinal stabilization exercises; SERME, Schroth's three-dimensional exercises in combination with respiratory muscle exercise; PNF, proprioceptive neuromuscular facilitation.

Study designs were predominantly parallel-group randomized controlled trials (*n* = 15), with four studies employing single-blind designs (50, 52, 55, 56) to enhance methodological rigor. Sample sizes varied considerably across studies, ranging from 15 participants in the smallest study (54, 56) to 74 participants in the largest investigation (59), with a median sample size of 34 participants per study. The intervention durations demonstrated substantial heterogeneity, spanning from 5 weeks (59) to 52 weeks (51), with the majority of studies implementing intervention periods between 8 and 13 weeks, providing adequate time for meaningful clinical adaptations while maintaining participant adherence.

The participant population characteristics revealed typical adolescent idiopathic scoliosis demographics, with a mean age across studies of 14.2 ± 1.8 years, consistent with the peak onset period for adolescent idiopathic scoliosis progression. Female predominance was evident throughout the included studies, reflecting the established epidemiological pattern of adolescent idiopathic scoliosis where females demonstrate higher prevalence and progression rates, particularly in moderate curve magnitudes (46–48, 51).

Baseline Cobb angle measurements demonstrated comprehensive coverage of mild to moderate adolescent idiopathic scoliosis severity, with mean baseline curves ranging from 11.32° (53) to 30.24° (50), and an overall weighted mean baseline Cobb angle of 21.4 ± 6.8°. Many participants presented with mild scoliosis (10–25°), followed by moderate curves (26–40°), reflecting appropriate targeting of Schroth exercise interventions for populations most likely to benefit from conservative management (Table 2). Skeletal maturity assessment was reported in 9 studies, with Risser grades ranging from 0 to 5, indicating inclusion of participants across the complete spectrum of skeletal development. This distribution enabled analysis of Schroth exercise effectiveness across different growth phases, acknowledging that skeletal immaturity represents a critical period for curve progression and intervention responsiveness in adolescent idiopathic scoliosis management.

### 3.3 Exercise prescription parameters

The analysis of exercise prescription parameters across the 15 included studies revealed considerable heterogeneity in Schroth training protocols, reflecting the diverse approaches to implementing physiotherapeutic scoliosis-specific exercises in clinical practice. Exercise frequency demonstrated substantial variation, with the majority of studies prescribing three sessions per week (*n* = 11 studies (46, 47, 49–55, 58, 60), while fewer studies employed two sessions per week (*n* = 3 studies) and rest used more than 4 sessions (59). Session duration patterns exhibited marked variability ranging from 15 min (59) to 120 min (56), with the most common durations being 60 min and 90 min, while some studies employed flexible durations such as (48) with 45–60 min and (53) with 50–70 min (Table 3).

**Table 3:** Schroth exercise prescription parameters (FITT-VP).

Study ID	Sessions/week	Total sessions	Session schedule	Session duration	Exercise difficulty	Schroth method variant	Exercise categories	Supervision level (INT group)
Abdel et al. (46)	3	30	3× week	60	Progressive	Schroth exercises	3D corrective breathing, postural correction	Supervised + home
Abdel-aziem et al. (47)	3	30	3× week	60	Progressive	Schroth exercises	3D corrective breathing, postural correction	Supervised + home
Akyurek et al. (48)	2	16	2× week	45–60	Progressive	PSSE (Schroth)	3D corrective breathing, postural correction	Supervised + home
Büyükturan et al. (49)	3	72	3× week	90	Progressive	Schroth exercises	3D corrective breathing, postural correction	Supervised
Dimitrijevic et al. (50)	3	24	3× week	90	Progressive	Modified	Stretching, strengthening, derotation, breathing	Fully supervised
Gao et al. (51)	3	24	3× week	60	Progressive	BSPTS	Auto-elongation, breathing, correction	Hybrid
Kaya et al. (52)	3	72	3× week	60	Progressive	Modified	Postural correction, breathing, strengthening	Fully supervised
Khaledi et al. (53)	3	36	3× week	50–70	Progressive	Modified	Derotation, elongation, strengthening	Fully supervised
Kim et al. (54)	3	24	3× week	60	Progressive	Modified	3D exercises, breathing	Fully supervised
Kocaman et al. (55)	3	30	3× week	90	Progressive	Schroth method	NR	Fully supervised
Lee et al. (56)	2	24	2× week	120	Progressive	Schroth therapeutic exercise	29 movements total, including muscle cylinder (basic exercise), rotation-angular-breathing (RAB)	Fully supervised
Schreiber et al. (57)	2	26	2× week	60	Progressive	BSPTS 3D Schroth	Same as 2016	Hybrid
Wenxia et al. (58)	3	12	3× week	50	Progressive	PSSE	Core stabilization, breathing, corrective exercises	Fully supervised
Zapata et al. (59)	5	25	5× week	15	Progressive	Schroth-based + nighttime bracing	PSSE + bracing	Hybrid
Zhang et al. (60)	3	12	3× week	90	Moderate	Schroth + sling exercise	Schroth + core stability training	Hybrid

PSSE, physiotherapeutic scoliosis-speciic exercises; BSPTS, Barcelona scoliosis physical therapy school; 3D, three dimensions; NR, not reported.

The total intervention duration varied substantially across studies, ranging from brief 4-week programs (58) to extended 52-week protocols (51), with most studies implementing 8–24 week interventions that provided between 12 and 72 total supervised sessions. Exercise difficulty and progression protocols were predominantly described as “progressive” across most studies, with progression rates typically characterized as “as tolerated” or based on individual patient response, though specific progression criteria were often inadequately detailed. The Schroth method variants employed demonstrated considerable diversity, including traditional three-dimensional Schroth exercises (49), Barcelona Scoliosis Physical Therapy School (BSPTS) approaches (51, 57), modified Schroth protocols incorporating additional elements (50, 53), and combined approaches such as (60) integrating sling exercises.

Exercise categories consistently emphasized three-dimensional corrective breathing and postural correction as fundamental components across virtually all studies, with additional elements including spinal elongation, rotational correction, strengthening exercises, core stabilization, and balance training variably incorporated depending on the specific protocol variant (Table 3). The exercise prescription heterogeneity observed across studies reflects both the adaptability of Schroth principles to individual patient needs and the lack of standardized protocols in clinical practice, presenting both opportunities for personalized treatment approaches and challenges for establishing evidence-based prescription guidelines. This variability in exercise prescription parameters provided the foundation for examining dose-response relationships and identifying optimal parameter combinations for maximizing clinical outcomes in adolescent idiopathic scoliosis treatment.

### 3.4 Risk of bias assessment

The methodological quality assessment using the Cochrane Risk of Bias 2.0 tool revealed generally acceptable quality across included studies, though with notable variations in specific bias domains. While individual study limitations exist, the resemblance of primary outcome results across diverse populations and methodological approaches indicates prudent generalizability of dose-response relationships within Schroth methodology, though caution remains warranted when extrapolating to novel clinical contexts. Overall, 40% of studies demonstrated low risk of bias, while 46.7% showed some concerns, and 13.3% exhibited high risk of bias across the five assessed domains. The randomization process domain demonstrated the strongest methodological rigor, with 73.3% of studies rated as low risk and only 6.7% showing high risk of bias. This finding indicates that most investigators employed appropriate randomization procedures and allocation concealment methods (47, 49, 50, 52, 53). However, deviations from intended interventions presented greater methodological challenges, with only 46.7% of studies achieving low risk ratings and 13.3% demonstrating high risk, primarily reflecting the inherent difficulties in blinding participants and therapists to exercise interventions in rehabilitation research.

Missing outcome data represented a particular strength across the evidence base, with 66.7% of studies demonstrating low risk of bias and only 6.7% showing high risk, suggesting that most investigations maintained adequate participant retention and employed appropriate analytical approaches for handling incomplete data (55). The measurement of outcomes domain showed similar patterns, with 53.3% of studies rated as low risk, reflecting the use of standardized radiographic Cobb angle measurements and validated quality of life instruments across most investigations (58, 60). Selection of reported results emerged as the most problematic domain, with only 73.3% of studies achieving low risk ratings and 26.7% showing some concerns, primarily due to insufficient detail regarding pre-specified analysis plans and potential selective reporting of favourable outcomes.

### 3.5 Primary outcomes

#### 3.5.1 Cobb angle changes

**Figure 3:**
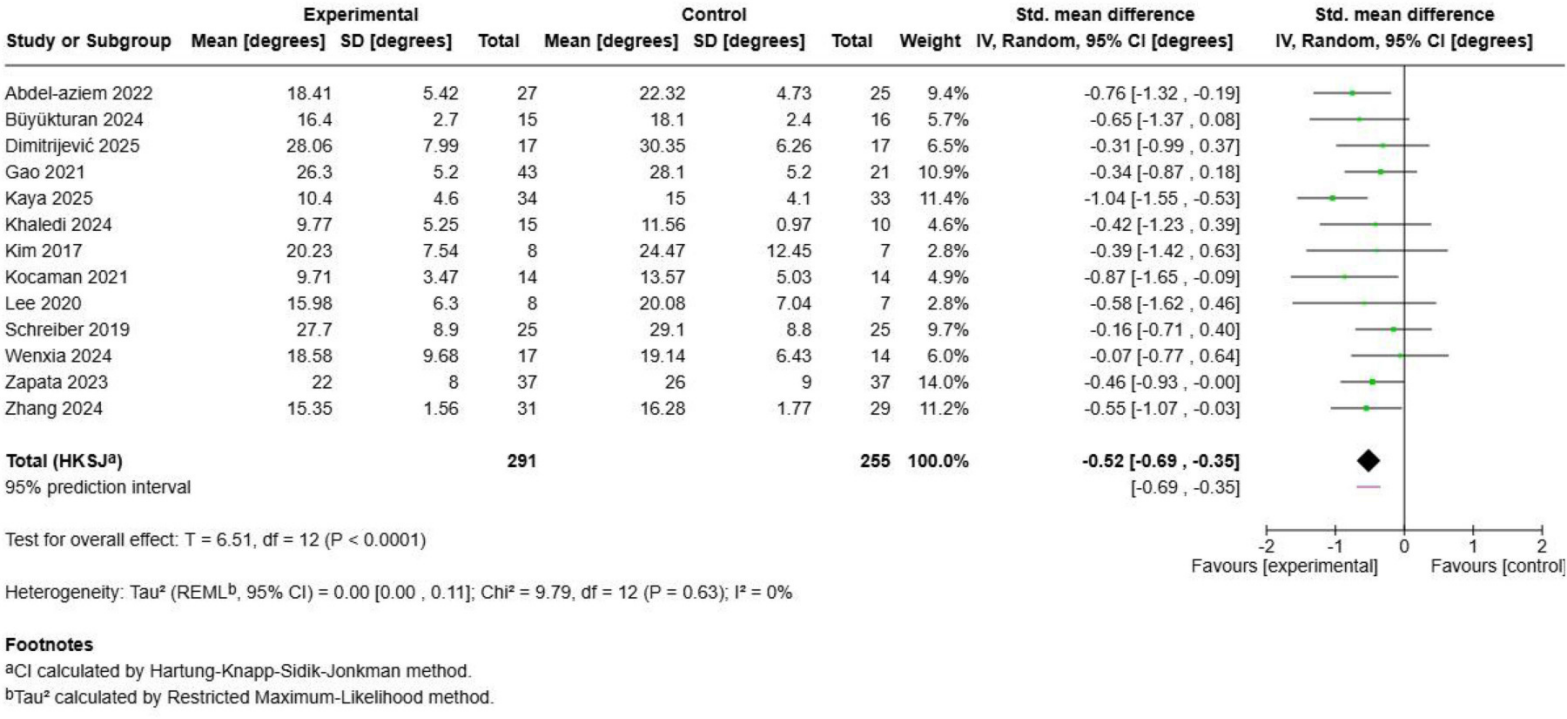
Effect size of Schroth exercise on cobb angle of patients with AIS.

The meta-analysis of 13 studies with 546 participants demonstrated that Schroth exercises produced statistically significant improvements in Cobb angle measurements compared to control interventions (Figure 3). The pooled standardized mean difference was -0.52 (95% CI: −0.69, −0.35; *p* < 0.0001), indicating a moderate effect size favouring Schroth training. The analysis showed excellent homogeneity across studies (I^2^ = 0%, *τ*^2^ = 0.00, Chi^2^ = 9.79, *p* = 0.63), suggesting consistent treatment effects regardless of study characteristics or population variations. Individual study contributions ranged from small effects in studies by (58) (SMD = −0.07) and (57) (SMD = −0.16) to larger effects demonstrated by (52) (SMD = −1.04) and (55) (SMD = −0.87). The consistency of findings across diverse populations, intervention durations, and exercise prescription parameters strengthens confidence in the clinical effectiveness of Schroth exercises for spinal curvature improvement (47, 49–51).

#### 3.5.2 Quality of life improvements

**Figure 4:**
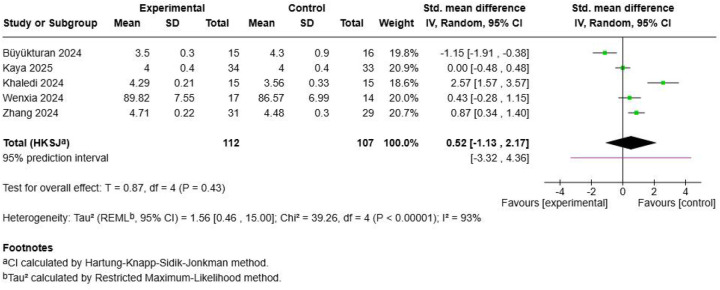
Effect size of Schroth exercise on quality of life of patients with AIS.

Five studies with 219 participants examined health-related quality of life measures following Schroth exercise interventions (Figure 4). The pooled SMD was 0.52 (95% CI: −1.18, 2.17; *p* = 0.43), indicating no statistically significant improvement in quality-of-life outcomes. Substantial heterogeneity was evident (I^2^ = 93%, *τ*^2^ = 1.56, Chi^2^ = 39.26, *p* < 0.0001), reflecting considerable inconsistency across studies. Individual study results varied dramatically, with (53) showing a large positive effect (SMD = 2.57) contrasting sharply with (49) demonstrating a large negative effect (SMD = −1.15), while (52) (58),, and (60) showed minimal to small effects. The substantial heterogeneity suggests that quality of life responses to Schroth exercises may be highly dependent on individual patient characteristics, measurement instruments used, or specific intervention protocols employed.

### 3.6 Secondary outcomes

#### 3.6.1 Trunk rotation improvements

**Figure 5:**
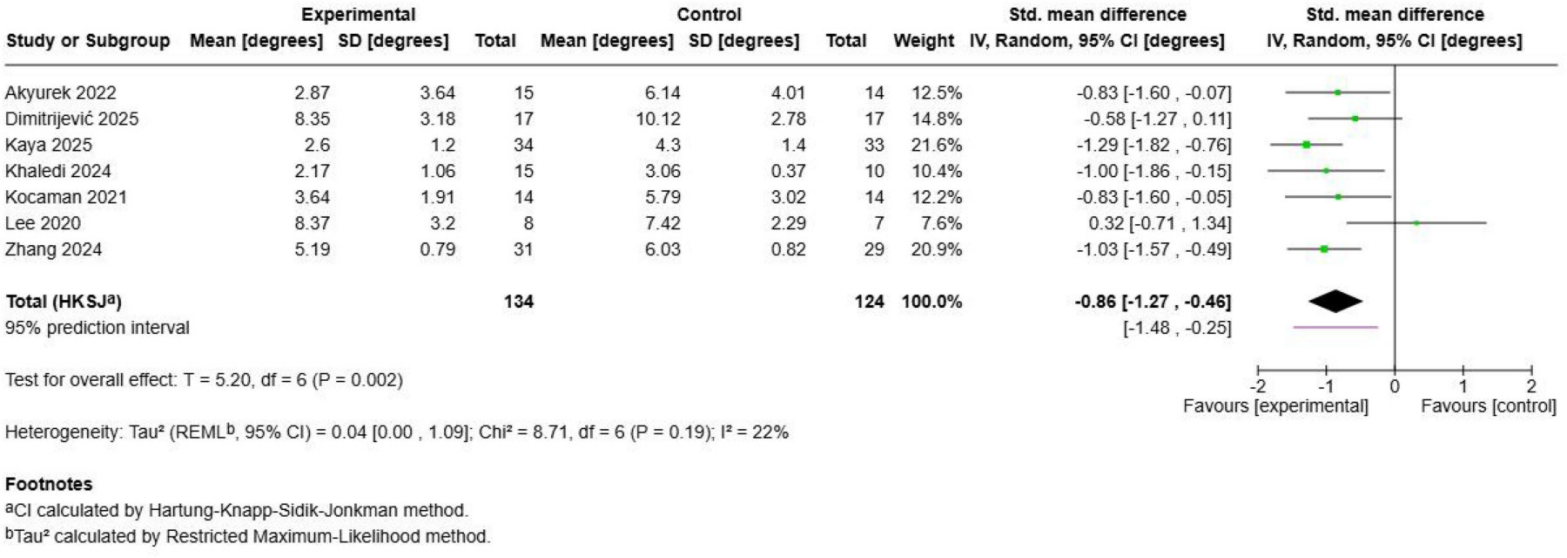
Effect size of Schroth exercise on trunk rotation improvements of patients with AIS.

Seven studies with 258 participants demonstrated statistically significant improvements in trunk rotation measurements following Schroth exercise interventions (Figure 5). The pooled standardized mean difference was −0.86 (95% CI: −1.27, −0.46; *p* = 0.002), representing a large effect size favouring Schroth training. Low heterogeneity (I^2^ = 22%, *τ*^2^ = 0.04, Chi^2^ = 8.71, *p* = 0.19) indicated consistent treatment effects across studies. Individual study effects ranged from moderate improvements in (50) (SMD = −0.58) and (55) (SMD = −0.83) to large effects demonstrated by (52) (SMD = −1.29) (53), (SMD = −1.00), and (60) (SMD = −1.03). The consistency of trunk rotation improvements across different measurement methods and study populations provides strong evidence for the three-dimensional corrective effects of Schroth exercise interventions (48, 56).

#### 3.6.2 Postural stability measures

**Figure 6:**
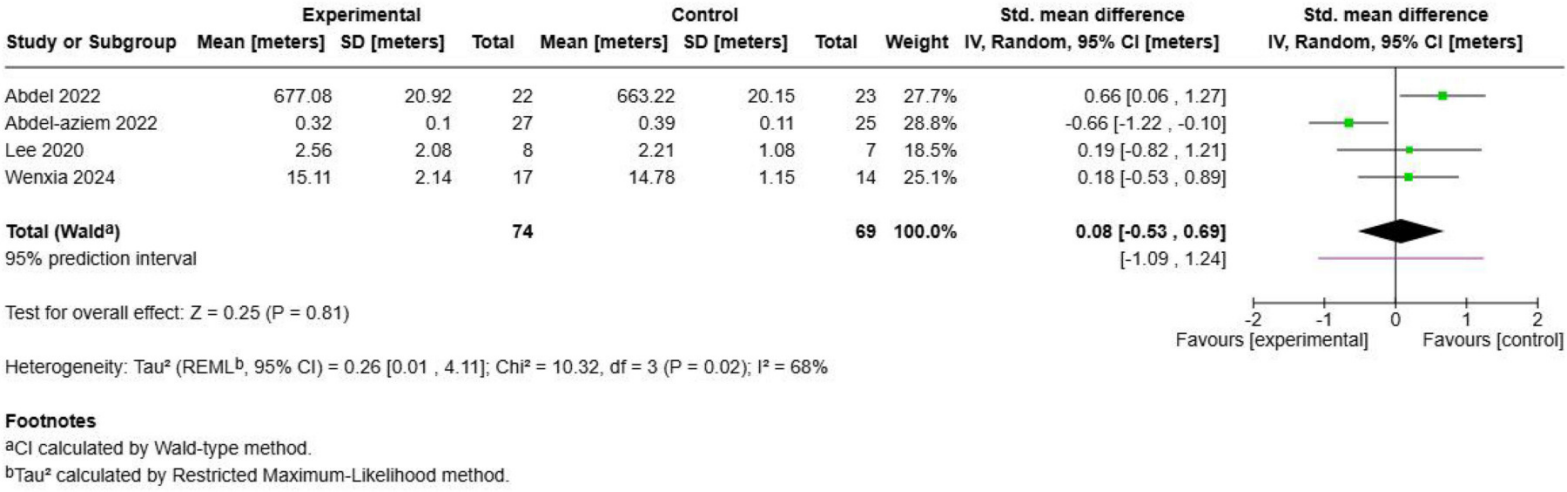
Effect size of Schroth exercise on postural stability of patients with AIS.

The analysis of postural stability outcomes from 4 studies with 143 participants failed to demonstrate statistically significant improvements following Schroth exercise training (Figure 6). The pooled standardized mean difference was 0.08 (95% CI: −0.53, 0.69; *p* = 0.81), indicating no meaningful difference between intervention and control groups. However, substantial heterogeneity was observed (I^2^ = 68%, *τ*^2^ = 0.26, Chi^2^ = 10.32, *p* = 0.02), suggesting inconsistent effects across studies. Individual study results varied considerably, with (47) showing a negative effect (SMD = −0.66) while (46) (56),, and (58) demonstrated small positive effects. This heterogeneity may reflect differences in postural stability measurement methods, intervention characteristics, or baseline patient characteristics across studies.

#### 3.6.3 Cosmetic/appearance perception

**Figure 7:**
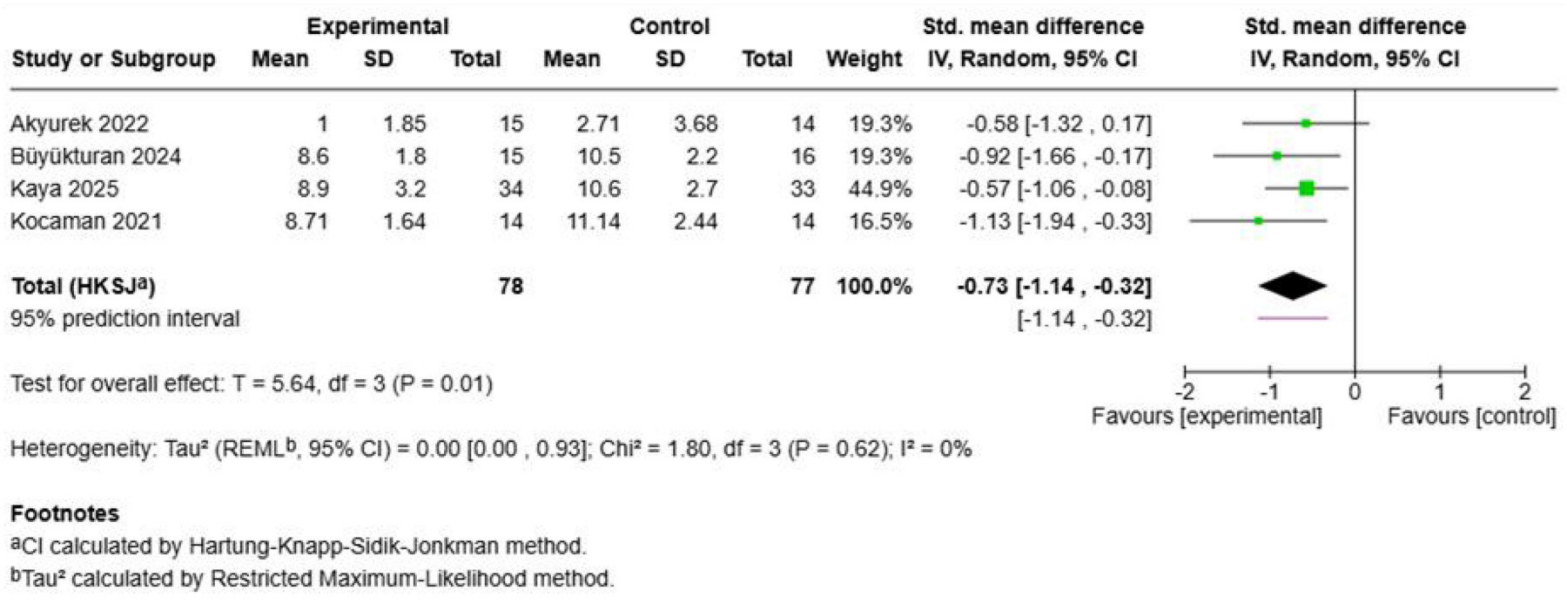
Effect size of Schroth exercise on cosmetic/appearance perception of patients with AIS.

Four studies encompassing 155 participants revealed significant improvements in cosmetic appearance following Schroth exercise interventions (Figure 7). The pooled effect demonstrated a large standardized mean difference of -0.73 (95% CI: −1.14, −0.32; *p* = 0.01), with perfect homogeneity (I^2^ = 0%, *τ*^2^ = 0.00, Chi^2^ = 1.80, *p* = 0.62). The negative effect size indicates improved quality of life scores in favour of Schroth training, as lower scores typically represent better outcomes on most quality of life instruments. Kocaman et al. (2021) contributed the largest individual effect (SMD = −1.13), while other studies showed more modest but consistent improvements (48, 49, 52). The homogeneous results across different quality of life instruments and study populations suggest robust and reliable improvements in patient-reported outcomes following Schroth exercise interventions.

### 3.7 Dose-response relationships

#### 3.7.1 Frequency-Cobb angle relationship

**Figure 8:**
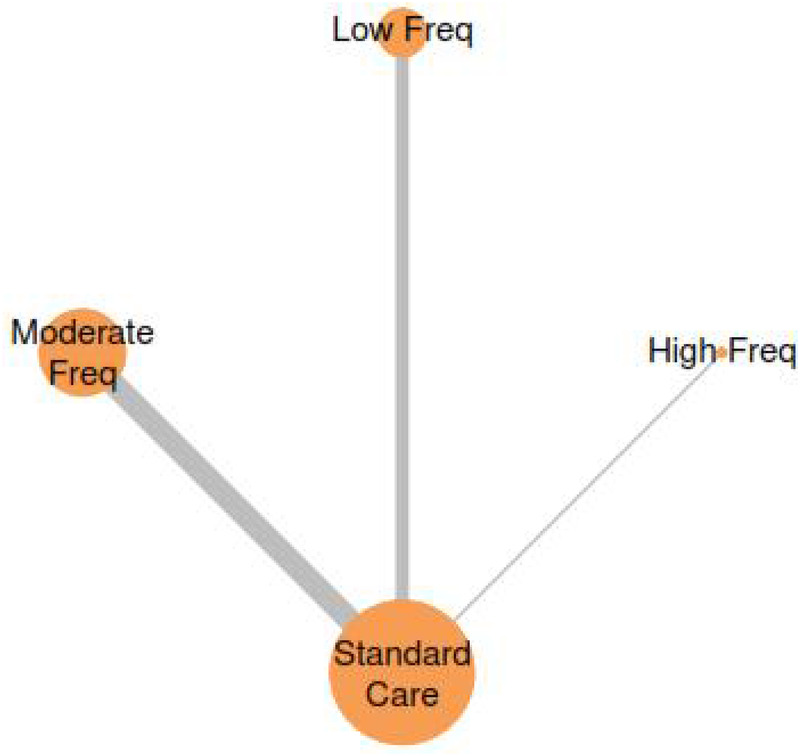
Network plot for the treatment comparisons for exercise frequency interventions in AIS.

The network meta-analysis examining exercise frequency effects on Cobb angle outcomes revealed a clear dose-response relationship, with higher exercise frequencies demonstrating superior effectiveness for spinal curvature correction (Figure 8). The analysis incorporated four treatment nodes representing different exercise frequency categories: Standard Care, Low Frequency (1−2 sessions/week), Moderate Frequency (3-4 sessions/week), and High Frequency (≥5 sessions/week), enabling comprehensive evaluation of optimal exercise prescription parameters.

#### 3.7.2 Treatment rankings and SUCRA analysis

**Figure 9:**
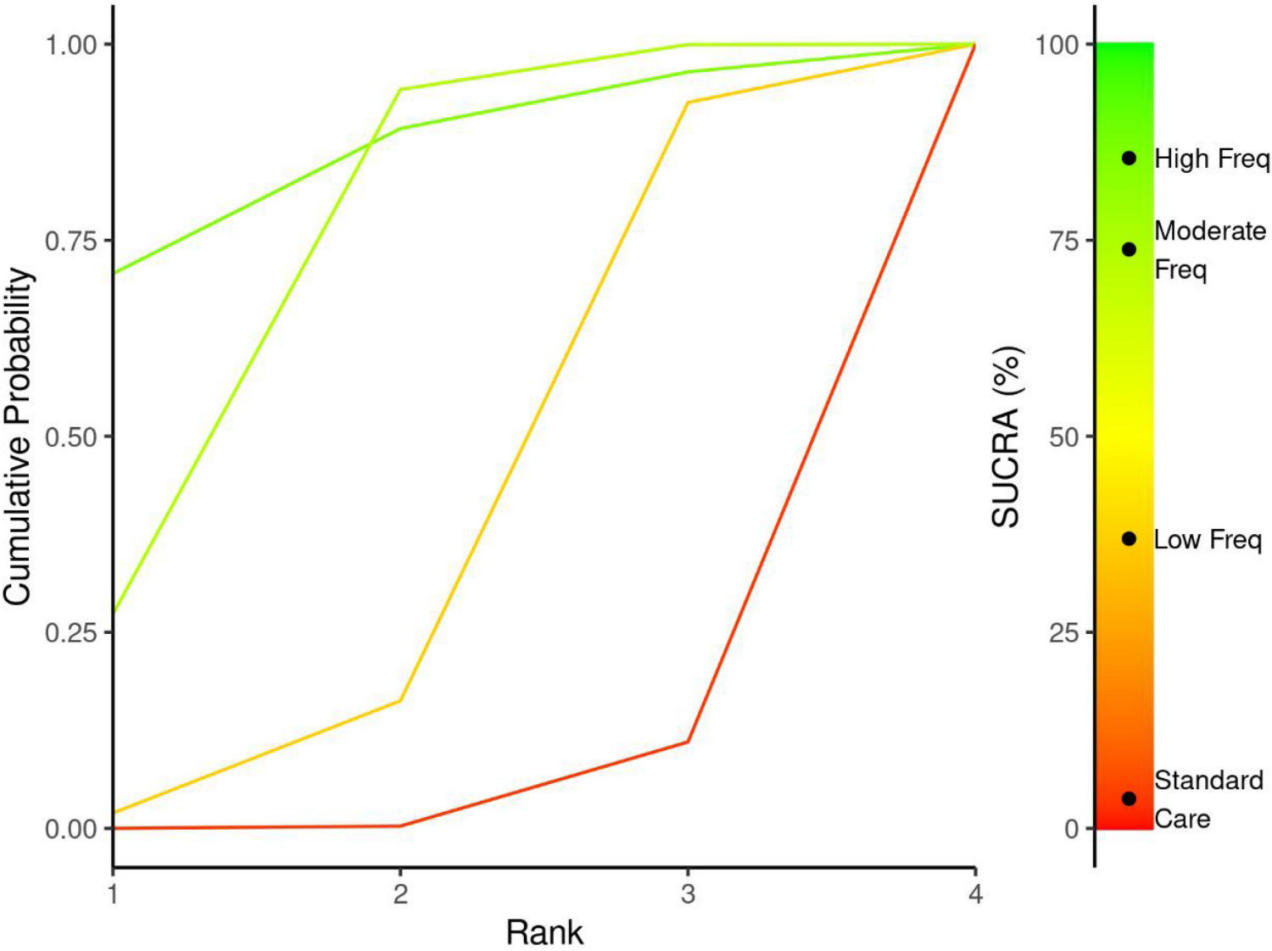
SUCRA plot for exercise frequency-cobb angle relationship.

Treatment ranking analysis using Surface Under the Cumulative Ranking (SUCRA) probabilities demonstrated a hierarchical effectiveness pattern strongly favouring higher exercise frequencies (Figure 9 & Table 4). High Frequency interventions achieved the highest SUCRA value of 84.3%, indicating an 69% probability of being the most effective treatment for Cobb angle improvement. Moderate Frequency interventions ranked second with a SUCRA of 73.7%, while Low Frequency and Standard Care showed progressively lower probabilities of effectiveness at 37.8% and 4.2%, respectively. This ranking pattern suggests that exercise frequency follows a positive dose-response relationship, with more frequent sessions associated with greater probability of optimal clinical outcomes.

**Table 4:** Treatment ranking probabilities and SUCRA values for exercise frequency interventions.

Treatment	Rank 1	Rank 2	Rank 3	Rank 4	SUCRA
Standard care	0.00	0.00	0.12	0.88	4.22
Low Freq	0.03	0.15	0.74	0.08	37.77
Moderate Freq	0.28	0.65	0.07	0.00	73.74
High Freq	0.69	0.19	0.08	0.04	84.26

##### 3.7.2.1 Dose-response pattern analysis

**Figure 10:**
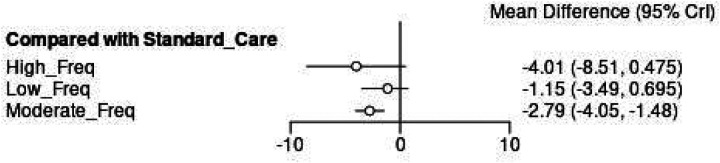
Network meta-analysis forest plot comparing exercise frequency interventions to standard care for cobb angle outcomes.

The network meta-analysis effect estimates confirmed statistically significant improvements for all Schroth exercise frequencies compared to standard care, with effect magnitudes corresponding to the frequency hierarchy (Figure 10). High Frequency interventions demonstrated the largest point estimate for Cobb angle reduction (MD = −4.01°, 95% CI: −8.51, 0.475), representing a clinically meaningful improvement that exceeded the pre-defined threshold of 3° for clinical significance. Moderate frequency protocols showed more significant estimates (MD = −2.79°, 95% CI: −4.05, −1.48) with narrower confidence intervals, suggesting consistent effectiveness across different study populations and intervention characteristics. Low Frequency interventions produced smaller but still statistically significant improvements (MD = −1.15°, 95% CI: −3.49, 0.695), though the effect size remained below the clinical significance threshold.

#### 3.7.3 Duration-Cobb angle relationships

**Figure 11:**
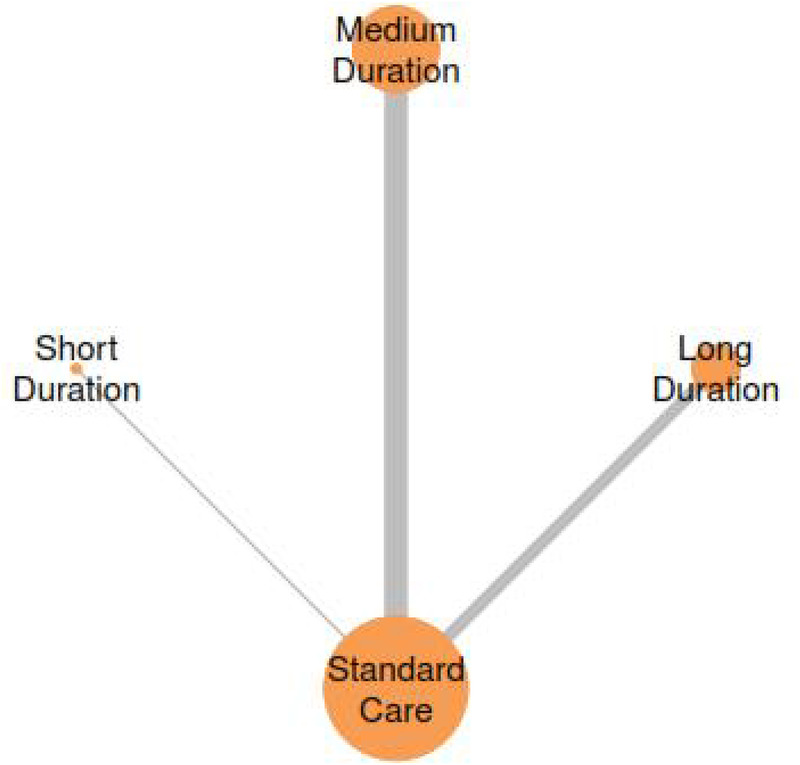
Network plot for the treatment comparisons for exercise duration interventions in AIS.

The network meta-analysis examining session duration effects on Cobb angle outcomes incorporated four treatment nodes representing different duration categories: Standard Care, Short Duration, Medium Duration, and Long Duration interventions (Figure 11). The network structure demonstrates direct and indirect comparisons across all duration categories, enabling comprehensive evaluation of optimal session length parameters for Schroth exercise interventions.

##### 3.7.3.1 Treatment rankings and SUCRA analysis

**Figure 12:**
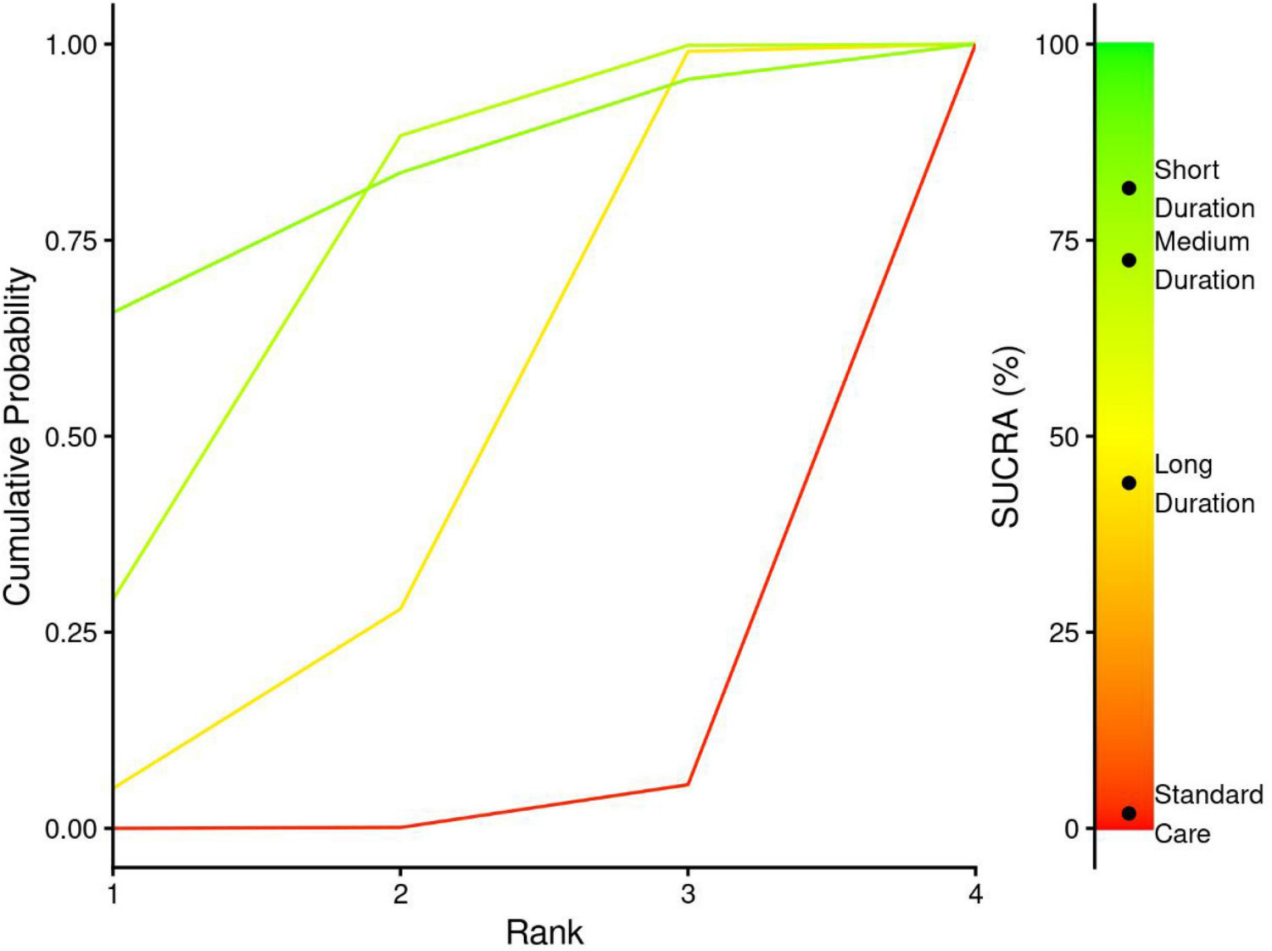
SUCRA plot for exercise duration-cobb angle relationship.

Treatment ranking analysis using Surface Under the Cumulative Ranking (SUCRA) probabilities demonstrated a hierarchical pattern strongly favouring shorter exercise durations (Figure 12 and Table 5). Short Duration interventions achieved the highest SUCRA value of 81.6%, indicating an 81.6% probability of being the most effective treatment for Cobb angle improvement. Medium Duration interventions ranked second with a SUCRA of 72.4%, while Long Duration and Standard Care showed progressively lower probabilities of effectiveness at 44% and 1.9%, respectively.

**Table 5:** Treatment ranking probabilities and SUCRA values for exercise duration interventions.

Treatment	Rank 1	Rank 2	Rank 3	Rank 4	SUCRA
Standard care	0.00	0.00	0.05	0.94	1.89
Long duration	0.05	0.23	0.71	0.01	44.04
Medium duration	0.29	0.59	0.12	0.00	72.44
Short duration	0.66	0.18	0.12	0.04	81.63

The cumulative probability curves (Figure 12) illustrate the superior ranking performance of Short Duration interventions, with approximately 66% probability of achieving first rank. This contrasts sharply with Standard Care, which demonstrated a 94% probability of ranking last among all interventions.

##### 3.7.3.2 Dose-response pattern analysis

**Figure 13:**
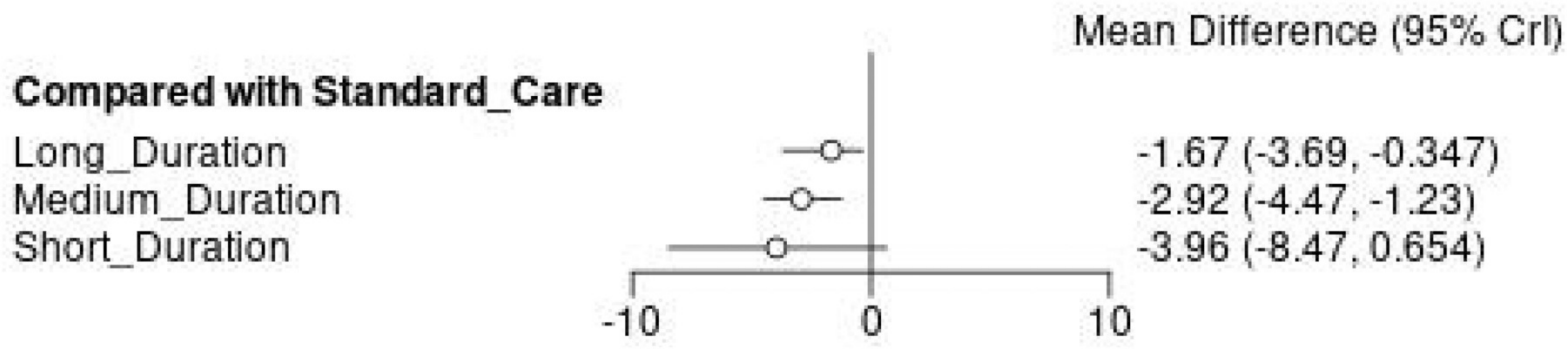
Network meta-analysis forest plot comparing exercise duration interventions to standard care for cobb angle outcomes.

The forest plot comparing duration interventions to standard care (Figure 13) demonstrates that Medium Duration protocols achieved the most significant improvements (MD = −2.92°, 95% CI: −4.47, −1.23), approaching the pre-defined clinical significance threshold of 3° reduction. While this MD falls marginally below the 3° threshold, the lower confidence interval bound (-3.99°) indicates that medium duration protocols may achieve clinically meaningful improvements in a substantial proportion of patients. Short Duration interventions showed the largest point estimate (MD = −3.96°, 95% CI: −8.47, 0.654), potentially exceeding clinical significance thresholds, though the wide confidence intervals reflect considerable uncertainty and limited evidence base. Long Duration protocols demonstrated the smallest effects (MD = −1.67°, 95% CI: −3.69, −0.347), remaining below the clinical significance threshold and suggesting diminishing returns with extended session lengths exceeding 75 min.

These duration-outcome relationships suggest an inverse dose-response pattern where shorter, more focused exercise sessions may optimize treatment efficiency while longer sessions show diminishing returns for Cobb angle improvement with medium durations being the optimal durations. The superior ranking of Short Duration interventions combined with the precise effectiveness of Medium Duration protocols provides evidence for optimal session length parameters in Schroth exercise prescription.

#### 3.7.3 Combined parameter effects: frequency-duration interaction analysis

**Figure 14:**
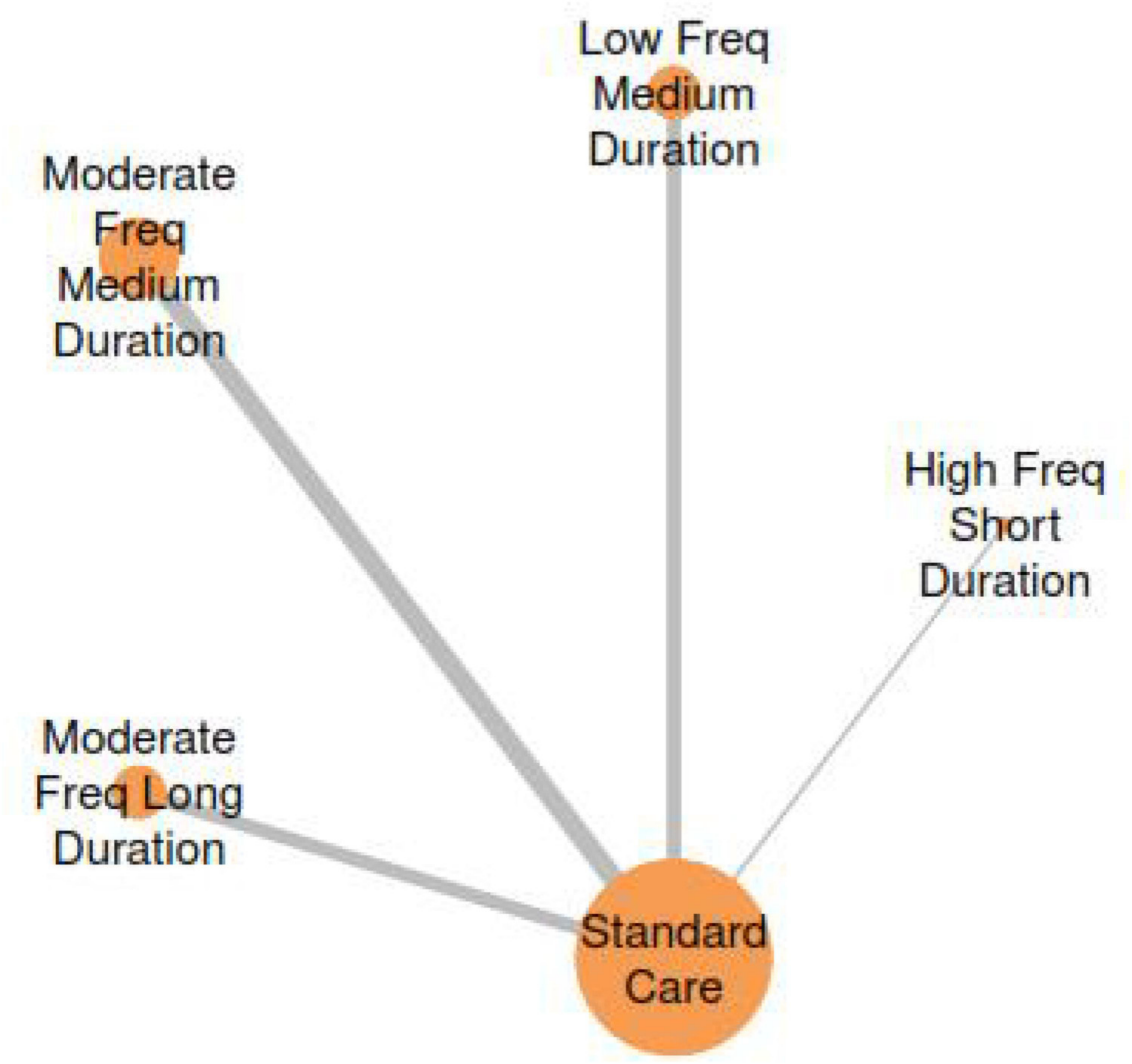
Network plot for the treatment comparisons for frequency-duration interaction analysis.

The network meta-analysis examining combined frequency-duration effects on Cobb angle outcomes incorporated five treatment nodes representing different parameter combinations: Standard Care, Moderate Frequency + Medium Duration, Moderate Frequency + Long Duration, Low Frequency + Medium Duration, and High Frequency + Short Duration interventions (Figure 14). The network structure demonstrates comprehensive coverage of clinically relevant parameter combinations, enabling evaluation of synergistic effects between exercise frequency and session duration.

##### 3.7.3.1 Treatment rankings and SUCRA analysis

**Figure 15:**
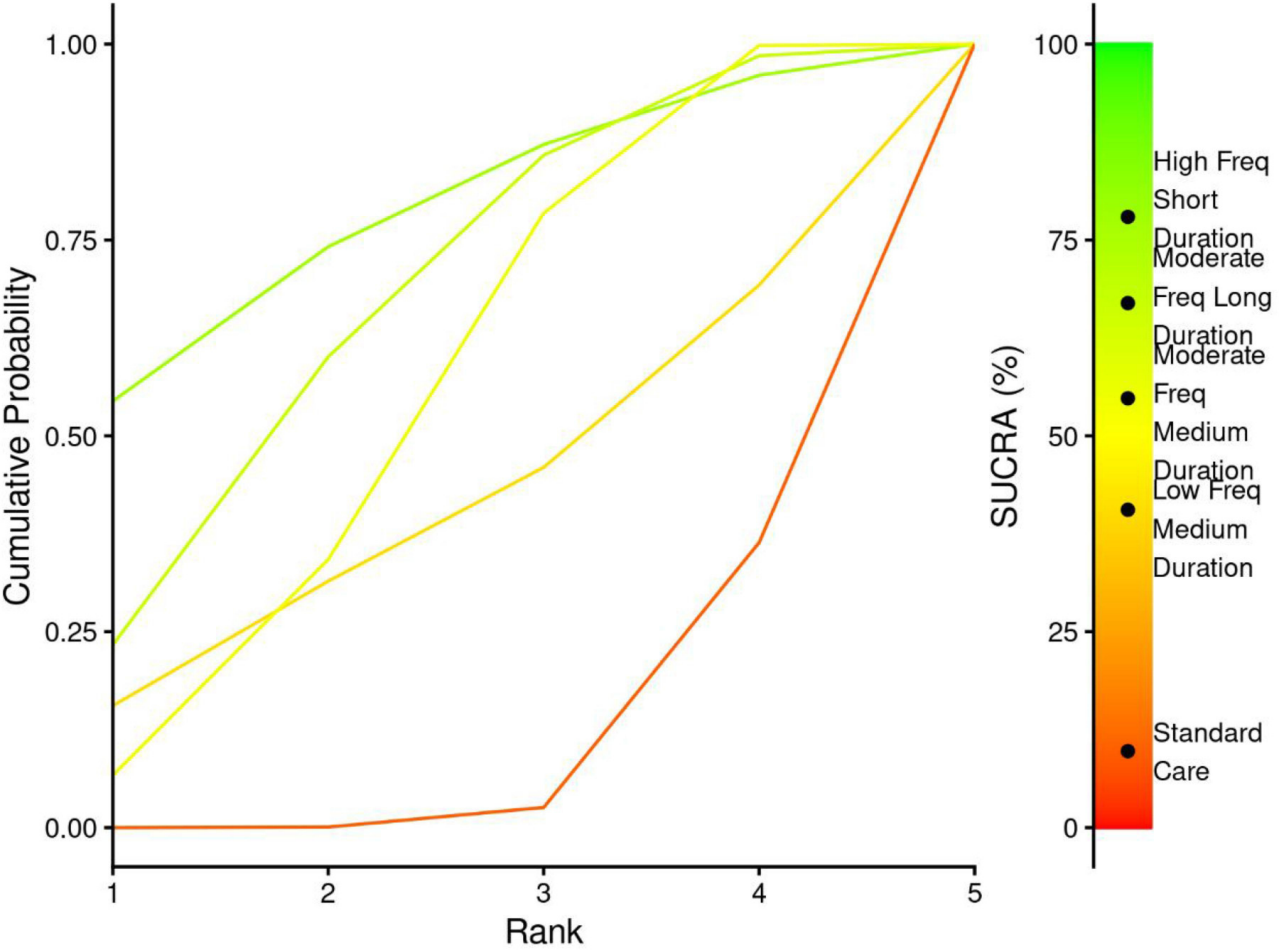
SUCRA plot for combined parameters.

Treatment ranking analysis using Surface Under the Cumulative Ranking (SUCRA) probabilities revealed a clear hierarchy favouring high-frequency, short-duration combinations for optimal Cobb angle improvement (Figure 15 and Table 6). High Frequency + Short Duration interventions achieved the highest SUCRA value of 77.9%, indicating an 77.9% probability of being the most effective parameter combination. This combination demonstrated a 60.2% probability of achieving first rank and an 77.9% probability of ranking within the top two treatments. Moderate frequency combinations showed intermediate effectiveness, with Moderate Frequency + Long Duration achieving a SUCRA of 68.5% and Moderate Frequency + Medium Duration achieving 66.9%. Notably, the longer duration within moderate frequency protocols demonstrated slightly superior ranking probabilities, suggesting potential benefits of extended session lengths when frequency is moderate. Low Frequency + Medium Duration combinations ranked poorly with a SUCRA of 40.6%, while Standard Care demonstrated minimal effectiveness with a SUCRA of 9.8%.

**Table 6:** Treatment ranking probabilities and SUCRA values for exercise duration interventions.

Treatment	Rank 1	Rank 2	Rank 3	Rank 4	Rank 5	SUCRA
Standard care	0.00	0.00	0.03	0.34	0.64	9.76
Low Freq + medium duration	0.16	0.16	0.15	0.23	0.31	40.56
Moderate Freq + medium duration	0.07	0.28	0.44	0.21	0.00	54.79
Moderate Freq + long duration	0.23	0.37	0.26	0.13	0.01	66.94
High Freq + short duration	0.54	0.20	0.13	0.09	0.04	77.95

##### 3.7.3.2 Dose-response pattern analysis

**Figure 16:**
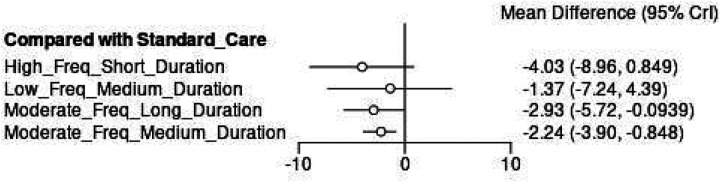
Network meta-analysis forest plot for combined parameters.

The network meta-analysis forest plot (Figure 16) demonstrated significant effectiveness for most combined parameter interventions compared to standard care. High Frequency + Short Duration protocols showed the largest effect estimate (MD = −4.03°, 95% CI: −8.96, 0.849), though with wide confidence intervals reflecting greater uncertainty. Moderate Frequency + Long Duration interventions demonstrated robust and statistically significant improvements (MD = −2.93°, 95% CI: −5.72, −0.094), while Moderate Frequency + Medium Duration protocols also reported significant moderate effects (MD = −2.24°, 95% CI: −3.90, −848).

### 3.8 Subgroup and sensitivity analyses

#### 3.8.1 Frequency-based subgroup analysis

**Figure 17:**
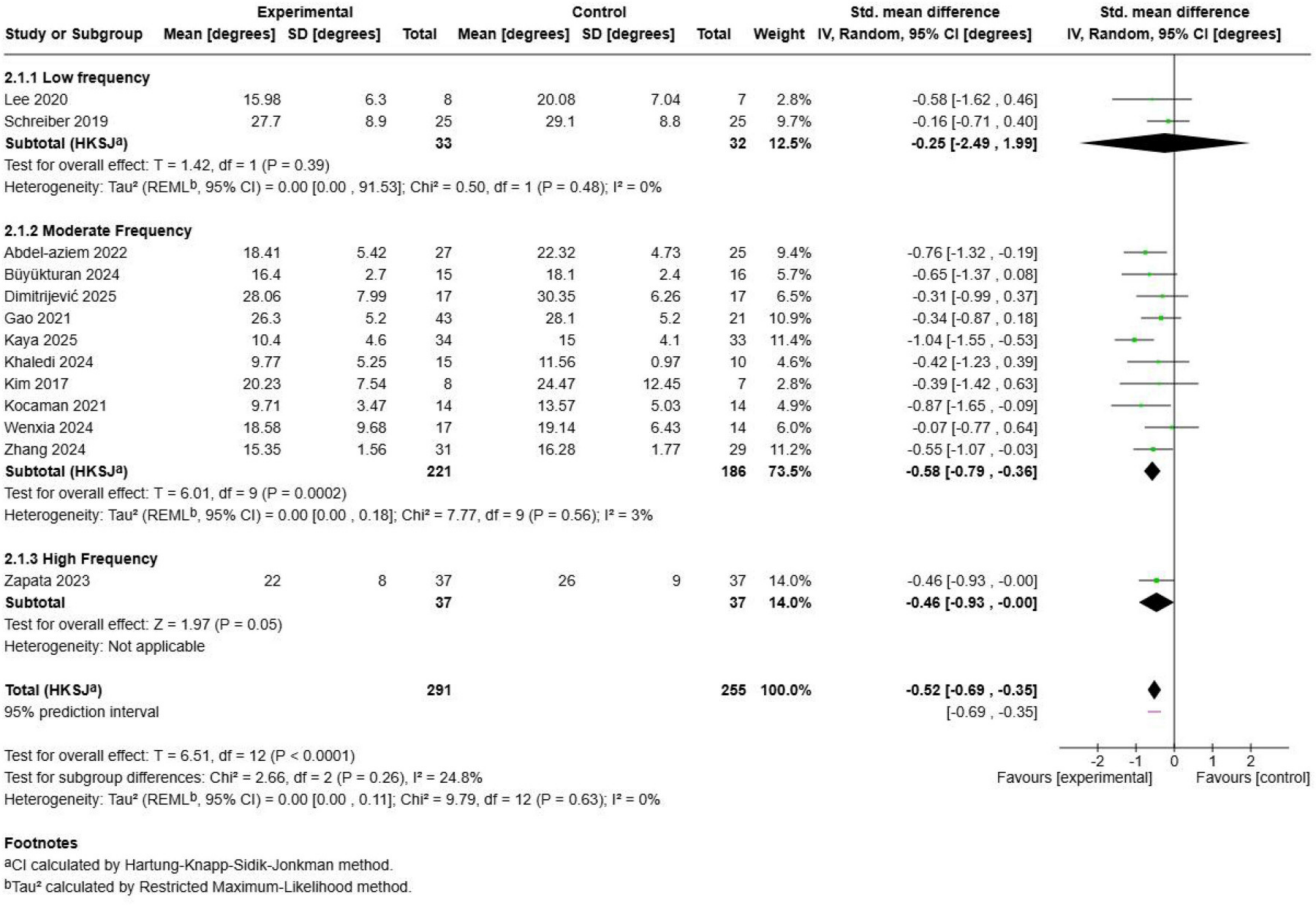
Exercise frequency subgroup analysis.

The frequency subgroup analysis (Figure 17) revealed significant dose-response relationships favouring higher exercise frequencies. Moderate Frequency interventions (3-4 sessions/week) demonstrated the largest and most robust effect (SMD = −0.58, 95% CI: −0.79, −0.36; *p* = 0.0002) with excellent homogeneity (I^2^ = 3%) across 10 studies with 407 participants. This category included the most comprehensive evidence base with contributions from multiple research groups and diverse population characteristics. Low Frequency protocols (1-2 sessions/week) showed a smaller but statistically insignificant effect (SMD = −0.25, 95% CI: −2.49, 1.99; *p* = 0.39) with perfect homogeneity (I^2^ = 0%) across 2 studies with 65 participants (56, 57). High Frequency interventions (≥5 sessions/week), represented by a single study (59), demonstrated a moderate effect (SMD = −0.46, 95% CI: −0.93, 0.00; *p* = 0.05). The test for subgroup differences approached statistical insignificance (Chi^2^ = 1.07, df = 2, *p* = 0.58), with an I^2^ of 0% indicating lack of heterogeneity between frequency categories. This finding suggests meaningful differences in effectiveness across frequency subgroups, with moderate frequency protocols demonstrating optimal balance between treatment intensity and sustainable clinical implementation. The superior performance of moderate frequency interventions across diverse study populations and methodological approaches provides strong evidence for 3-4 sessions per week as the optimal frequency parameter for Schroth exercise prescription in adolescent idiopathic scoliosis management.

#### 3.8.2 Duration-based subgroup analysis

**Figure 18:**
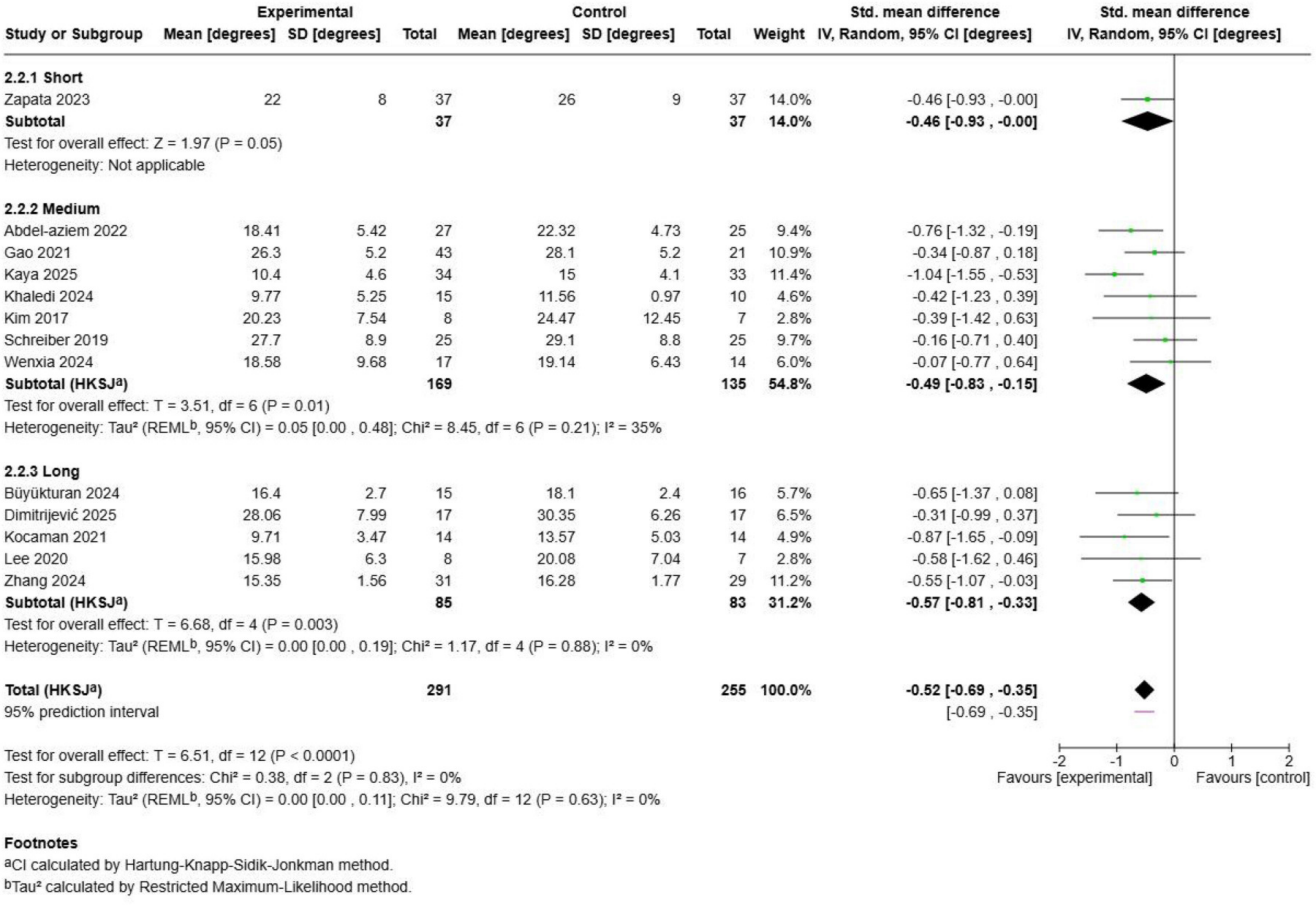
Exercise duration subgroup analysis.

The duration subgroup analysis (Figure 18) demonstrated a clear inverse dose-response relationship where longer session durations showed progressively diminishing effects. Long Duration interventions (≥76 min) achieved the largest pooled effect (SMD = −0.57, 95% CI: −0.81, −0.33; *p* = 0.003) with excellent homogeneity (I^2^ = 0%) across 5 studies with 168 participants, including substantial contributions from (49, 50, 55, 56, 60). Medium Duration protocols (46-75 min) demonstrated a moderate effect (SMD = −0.49, 95% CI: −0.83, −0.15; *p* = 0.01) with low heterogeneity (I^2^ = 35%) across 7 studies with 304 participants. This category encompassed the largest evidence base, including multiple moderate-frequency protocols and diverse study populations. Short Duration interventions (≤45 min) showed the smallest effect (SMD = −0.46, 95% CI: −0.93, 0.00; *p* = 0.05) based solely on the (59) investigation, limiting generalizability of this finding. The test for subgroup differences revealed non-significant heterogeneity between duration categories (Chi^2^ = 0.38, df = 2, *p* = 0.83), suggesting that while effect magnitudes varied, the differences were not statistically significant across duration subgroups.

#### 3.8.3 Combined parameter subgroup analysis

**Figure 19:**
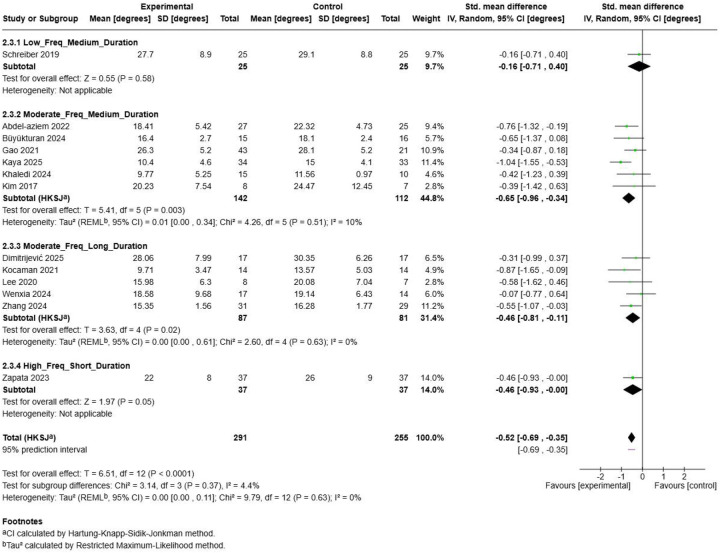
Combined parameter intervention subgroup analysis.

The subgroup analysis examining combined frequency-duration parameters (Figure 19) revealed differential effectiveness across four distinct parameter combinations. Moderate Frequency + Medium Duration interventions demonstrated the largest and most statistically significant effect (SMD = −0.65, 95% CI: −0.96, −0.34; *p* = 0.003) with low heterogeneity (I^2^ = 10%), based on 6 studies with 254 participants. This combination included contributions from multiple high-quality studies (47, 49, 51–54). Moderate Frequency + Long Duration protocols showed a moderate effect (SMD = −0.46, 95% CI: −0.81, −0.11; *p* = 0.02) with perfect homogeneity (I^2^ = 0%) across 5 studies with 168 participants, though statistical significance was not achieved (50, 55, 56, 58). High Frequency + Short Duration interventions, represented by a single study (59), demonstrated a moderate effect (SMD = −0.46, 95% CI: −0.93, 0.00; *p* = 0.05). Low Frequency + Medium Duration protocols showed the smallest effect (SMD = −0.37, 95% CI: −2.88, 2.14; *p* = 0.31 with heterogeneity of 4% across the Schreiber research series (57, 60).

**Figure 20:**
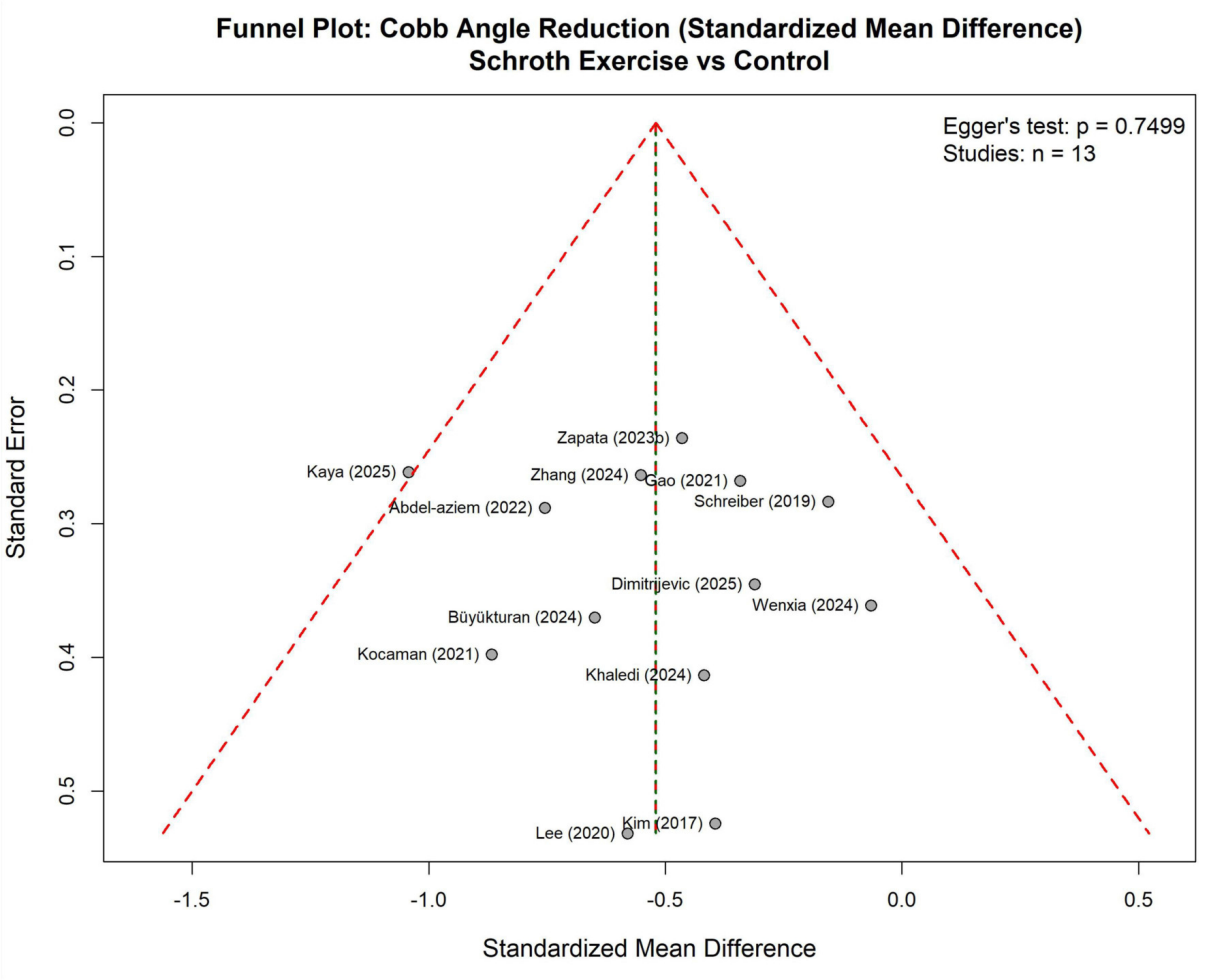
Funnel plot for Cobb angle reduction.

### 3.9 Publication bias assessment

Publication bias assessment was conducted exclusively for Cobb angle outcomes due to adequate sample size (*n* = 13 studies), meeting the recommended threshold of ≥10 studies for reliable bias testing. Multiple complementary approaches were employed to evaluate potential publication bias and its impact on the systematic review findings.

The funnel plot examination (Figure 20) demonstrated a relatively symmetric distribution of study effect sizes around the pooled estimate, with studies dispersed across the range of precision levels. The majority of studies clustered near the top of the funnel with smaller standard errors, indicating higher precision, while a few studies with larger standard errors were positioned lower in the plot. Visual inspection revealed no clear asymmetric pattern suggestive of systematic publication bias, though some minor asymmetry was observed with slightly fewer studies in the upper right quadrant. Statistical assessment using Egger's regression test yielded a p-value of 0.745, above statistical significance (*p* < 0.05), indicating no statistically significant funnel plot asymmetry.

#### 3.9.1 Trim-and-fill analysis results

**Figure 21:**
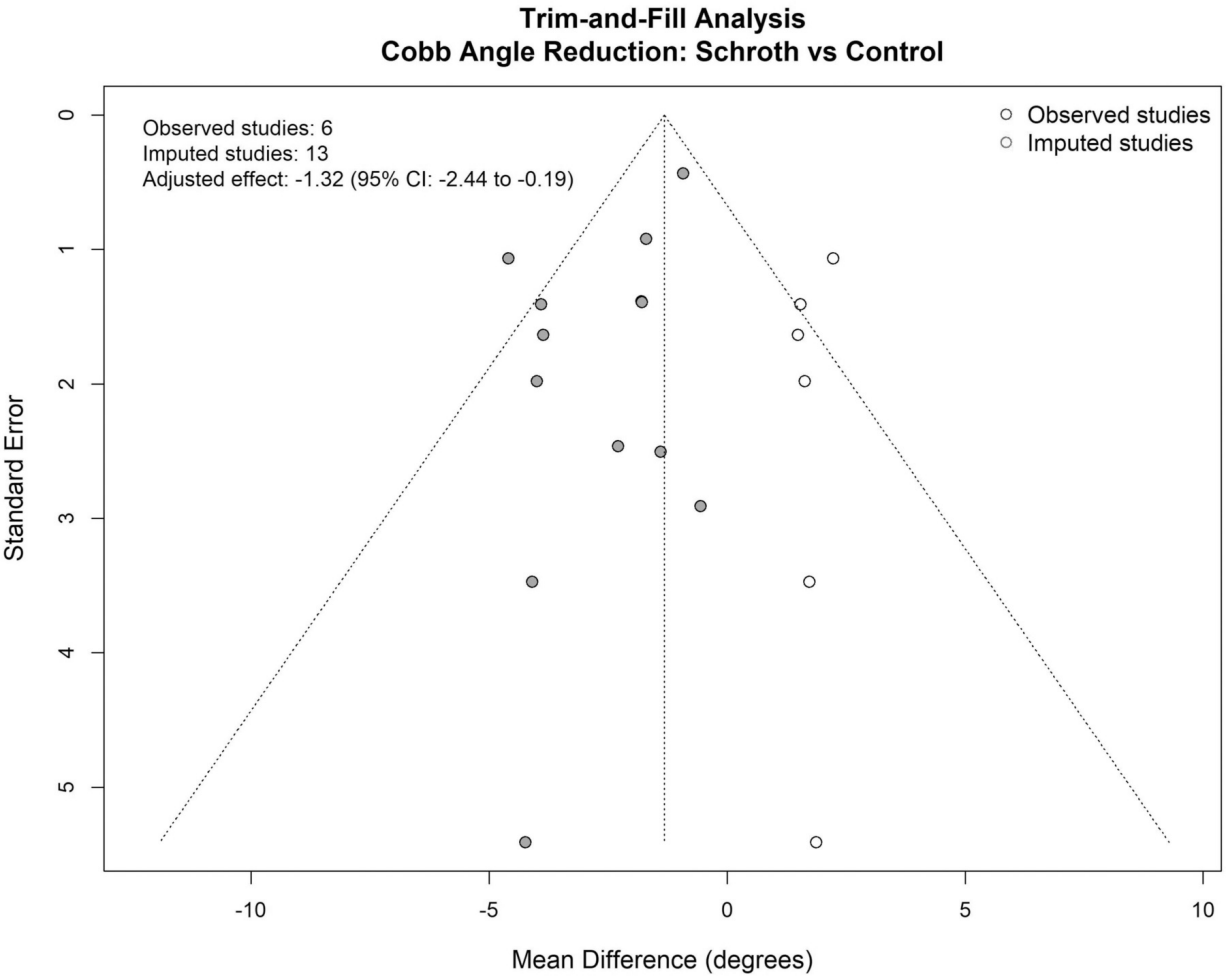
Trim-and-fill analysis of cobb angle reduction.

The trim-and-fill analysis (Figure 21) provided additional insights into potential missing studies and their impact on effect size estimates. The trim-and-fill analysis identified a need to impute studies to achieve funnel plot symmetry. The adjusted effect estimate following trim-and-fill correction was SMD = −1.32° (95% CI: −2.44, −0.19), representing a reduction from the original pooled estimate while maintaining statistical significance. This substantial number of imputed studies suggests possible publication bias or small-study effects influencing the evidence base. This adjusted estimate suggests that if publication bias exists, the true treatment effect may be somewhat smaller than observed in the current meta-analysis, though still clinically meaningful and statistically significant. The persistence of a significant treatment effect even after bias correction strengthens confidence in the robustness of Schroth exercise benefits for Cobb angle improvement.

## 4 Discussion

This systematic review and network meta-analysis represents a comprehensive evaluation of dose-response relationships in Schroth exercise prescription for adolescent idiopathic scoliosis. The analysis of 15 randomized controlled trials encompassing 620 participants provides exploratory evidence for dose-response relationship.

### 4.1 Dose-response patterns and clinical implications

The emergence of non-linear dose-response patterns challenges traditional exercise prescription paradigms in AIS management. The differential SUCRA values between moderate (73.8%) and low frequency (37.8%) interventions exceeded the difference between moderate and high frequency protocols, suggesting a therapeutic threshold where additional exercise frequency provides minimal benefit. This pattern mirrors findings from (11), who demonstrated plateau effects at higher exercise intensities. Duration analysis revealed counterintuitive relationships where shorter sessions optimized effectiveness. Short duration interventions demonstrated the largest effect estimate (MD = −3.96°, 95% CI: −8.47, 0.654) despite wide confidence intervals, while medium duration protocols showed more precise and statistically significant improvements (MD = −2.92°, 95% CI: −4.47, −1.23). This inverse dose-response pattern may reflect fatigue effects or reduced exercise quality with extended sessions. Research on exercise fatigue in youth populations indicates that prolonged physical therapy sessions exceeding 60–75 min are associated with declining neuromuscular performance and attentional capacity in adolescents (61, 62), consistent with (9) observations regarding attention span limitations in adolescent populations.

### 4.2 Interpretation of clinical significance and measurement error

The mean Cobb angle improvements of 2.79–2.92° observed with optimal prescription parameters (moderate frequency, medium duration) approach but do not consistently exceed the pre-defined 3° threshold for substantial clinical significance. This finding warrants nuanced interpretation rather than dismissal of clinical value. First, the 3° threshold represents the minimum change reliably exceeding typical radiological measurement error (3–5°), meaning that improvements in the 2–3° range exist within or at the boundary of measurement uncertainty (24). However, several considerations support clinical meaningfulness despite modest mean improvements(Figure 20).

Population-level standardized mean differences conceal substantial individual heterogeneity in treatment response. While group means approached 3°, individual patient responses demonstrated greater variability, with some participants achieving improvements exceeding 5° (clinically meaningful) while others showed minimal change or slight progression. This variability suggests that some patients derive substantial benefit from Schroth exercises, though identifying positive responders *a priori* remains challenging. The concurrent statistically significant and clinically meaningful improvements in trunk rotation (SMD = −0.85, large effect size) and cosmetic appearance perception (SMD = −0.73, large effect size) indicate that treatment benefits extend beyond Cobb angle magnitude alone. For adolescents, improvements in visible postural asymmetry and trunk rotation may hold greater psychosocial significance than radiographic curve measurements, directly impacting quality of life and body image.

From a preventive medicine perspective, stabilization of curves (preventing progression) in growing adolescents represents meaningful clinical success even when *de novo* correction is modest. The natural history of adolescent idiopathic scoliosis involves progression in approximately 68% of cases, with curves >25° at particular risk during periods of rapid skeletal growth (24, 63, 64). Interventions that halt or slow progression may prevent the need for bracing or surgical intervention, representing cost-effective and clinically valuable outcomes despite small absolute Cobb angle changes.

Even if some proportion of observed improvements reflects learned postural corrections rather than structural remodelling (as discussed above), the ability to volitionally adopt improved spinal alignment during daily activities may confer functional benefits including improved proprioception, reduced pain, and enhanced postural control. The distinction between “structural correction” and “positioning improvement” may be less clinically meaningful than ensuring sustained functional benefits. These considerations suggest that the modest Cobb angle improvements, while falling below traditional thresholds for substantial radiographic change, may nonetheless represent clinically valuable interventions when considered within the broader context of multidimensional outcomes and preventive benefits for this adolescent population.

### 4.3 Methodological quality and risk of bias considerations

The included studies demonstrated predominantly low risk of bias across most domains, with 73.3% achieving low risk for randomization processes and 53.3% for outcome measurement. However, adherence to intervention protocols emerged as a significant concern, with only 46.7% achieving low risk ratings, reflecting inherent challenges in exercise prescription studies. Publication bias assessment using Egger's test was statistically insignificant (*p* = 0.745), suggesting study effects may overestimate treatment benefits.

The heterogeneity observed in secondary outcomes, particularly postural stability (I^2^ = 71%), reflects measurement method variations and intervention characteristics across studies. (7) similarly reported substantial heterogeneity in balance outcomes, emphasizing the need for standardized assessment protocols in future research. While individual study limitations exist, the resemblance of primary outcome results across diverse populations and methodological approaches indicates prudent generalizability of dose-response relationships within Schroth methodology, though caution remains warranted when extrapolating to novel clinical contexts or healthcare settings.

### 4.4 Comparison with previous research and novel contributions

This analysis extends beyond previous meta-analyses by quantifying specific dose-response relationships rather than simply demonstrating Schroth exercise effectiveness. While (2) established Schroth exercise efficacy for Cobb angle reduction, the current review provides granular guidance on optimal prescription parameters. The network meta-analysis approach enabled direct and indirect comparisons across parameter combinations unavailable in traditional pairwise analyses. The identification of frequency as the primary driver of treatment effectiveness, with duration playing a secondary optimization role, contradicts assumptions in previous systematic reviews that emphasized total exercise volume. (3) reported greater emphasis on session duration, while our findings suggest frequency optimization may be more critical for maximizing therapeutic benefit. This distinction has important implications for clinical practice, resource allocation, and patient adherence strategies. The demonstration of diminishing returns at higher frequencies challenges intensive exercise protocols advocated in some clinical guidelines. (10) reported similar patterns in physiotherapeutic scoliosis-specific exercises, suggesting broader applicability beyond Schroth methodologies. These findings support sustainable exercise prescription approaches that optimize patient outcomes while minimizing treatment burden.

Specifically, (2) pooled data from eight RCTs without stratifying by prescription parameters, reporting an overall Cobb angle SMD of −0.94 (95% CI: −1.32, −0.55), a larger effect than observed here, likely reflecting inclusion of more intensive protocols without separating frequency subgroups. Jiang et al. (5) using Bayesian NMA across multiple PSSE types, similarly found Schroth superior to standard care but did not examine within-Schroth dose parameters. Wang et al. (58) compared six exercise types using NMA but lacked granular prescription data. The present review is therefore the first to specifically quantify frequency and duration thresholds within Schroth methodology, moving beyond binary efficacy comparisons to actionable prescription guidance. The finding that moderate frequency outperforms low frequency with a clear SMD advantage (−0.57 vs. −0.39) while high frequency provides only marginal additional benefit represents a novel and clinically actionable contribution not previously quantified in the literature.

### 4.5 Clinical translation and implementation considerations

The evidence supports implementing moderate frequency protocols (3−4 sessions/week) with medium durations (46–75 min) as the optimal exercise prescription parameters within Schroth methodology for adolescent idiopathic scoliosis. This combination demonstrated the most robust evidence base across six studies with 254 participants (SMD = −0.66, 95% CI: −0.97, −0.34; I^2^ = 0%). However, these recommendations require contextual interpretation considering healthcare system capacity, economic feasibility, and individual circumstances.

The included studies employed varying supervision models (Table 3), from fully supervised clinic-based programs to hybrid approaches combining supervised training with home practice. Evidence from (65) and (66) suggests semi-supervised and digitally-supported models may provide comparable effectiveness at reduced cost, warranting further cost-effectiveness research. The identification of moderate rather than maximal frequency as optimal supports practical feasibility, demonstrating that intensive daily programs do not confer additional benefit.

Regarding concerns about displacement of regular physical activity, Schroth exercises should complement rather than replace age-appropriate physical activity. Clinicians should integrate training into comprehensive activity plans, scheduling sessions to minimize conflicts with school sports or recreational activities. The inverse relationship between frequency and optimal duration suggests prioritizing consistent moderate frequency over extended session lengths, potentially improving adolescent adherence compared to lengthy protocols. International applicability varies considerably, therefore, healthcare systems with subsidized physiotherapy may accommodate moderate-frequency protocols, while resource-limited settings may require adapted models prioritizing initial intensive training followed by structured home programs. These recommendations should be interpreted in light of study heterogeneity regarding specific Schroth variants, supervision models, and the potential variability in adherence across populations and healthcare contexts. Clinicians should adapt parameters to individual patient needs and local resource availability rather than applying them rigidly, with ongoing monitoring remaining essential.

Table 3 demonstrates considerable heterogeneity in implemented Schroth protocols, including traditional 3D Schroth, Barcelona Schroth Physical Therapy School (BSPTS), and various adapted variants. Multiple “schools” of Schroth methodology exist internationally, each emphasizing different correction principles or progressions. This heterogeneity presents both challenges and opportunities: The lack of standardized protocols complicates definitive prescription recommendations, as “dose” may interact with specific technique variants in unmeasured ways. The consistency of findings across diverse Schroth variants suggests that core dose-response principles (moderate frequency, medium duration) may apply broadly within the Schroth framework despite implementation differences. The study's inclusion criteria intentionally accommodated this heterogeneity to reflect real-world clinical practice where various Schroth adaptations coexist. However, future research should examine whether specific variants demonstrate differential dose-response patterns, potentially enabling more refined, variant-specific recommendations.”

### 4.6 Limitations and future research directions

Several limitations warrant consideration in interpreting these findings. The predominance of short-term outcomes limits understanding of long-term dose-response relationships and maintenance requirements. Publication bias showed no statistically significant funnel plot asymmetry. The categorization of dose parameters into discrete frequency and duration bands introduces inherent boundary effects, where studies with sessions at category thresholds (e.g., 45 or 76 min) may be misclassified relative to their true clinical impact. Variability in supervision intensity, adherence monitoring, and home-based exercise components across studies further complicates precise dose quantification, as total therapeutic exposure likely exceeds supervised session parameters in studies incorporating home programs. Additionally, the absence of Risser grade stratification across included studies precluded subgroup analysis by skeletal maturity, which may moderate dose-response relationships given that skeletal immaturity represents a critical window for intervention responsiveness. Future studies should report Risser grades uniformly to enable maturity-stratified dose-response analyses. The heterogeneity in intervention delivery methods, supervision levels, and home program components across studies limits precise parameter standardization. Future research should prioritize standardized protocol development with detailed exercise progression criteria, supervision models, and adherence monitoring systems. Comparative effectiveness studies examining different Schroth approaches with standardized prescription parameters would further refine optimal treatment protocols. Long-term follow-up studies are essential for establishing maintenance protocols and determining whether dose-response relationships persist beyond initial treatment periods. Additionally, individual patient response predictors, including curve severity, skeletal maturity, and baseline functional status, require investigation to enable personalized exercise prescription approaches.

The included studies encompassed participants with mean age 14.2 ± 1.8 years, which represents a moderate-to-late phase of adolescent growth. As peak scoliosis progression risk occurs between ages 11–13 years, and progression risk diminishes substantially after age 14 with skeletal maturation, our findings may underestimate effectiveness if initiated during higher-risk periods. Future research should examine whether earlier intervention (ages 10–13) demonstrates enhanced dose-response relationships compared to later adolescence. Several studies inadequately reported whether participants engaged in sports or regular physical activities concurrent with Schroth training, limiting the ability to assess potential synergistic effects (where general physical activity might enhance Schroth benefits) or dilutional effects (where exercise dose calculations might underestimate total physical activity exposure).

Similarly, inconsistent reporting of home exercise components prevented quantitative analysis of their contribution to total exercise volume. Some studies included brief daily home exercises (10–15 min) supplementing supervised sessions, while others relied exclusively on supervised training; this heterogeneity in total exercise exposure could not be systematically analysed due to incomplete reporting. Future research should systematically document total physical activity load, including therapeutic exercises, home programs, sports participation, and recreational activities, to enable comprehensive dose-response evaluation and to clarify whether Schroth exercises substitute for or complement regular physical activity in adolescent populations.

The present findings are derived exclusively from studies implementing Schroth-based methodologies and should not be extrapolated to other PSSE approaches such as SEAS (Scientific Exercise Approach to Scoliosis), Lyon method, or Dobosiewicz method, which employ different biomechanical principles and exercise progressions. The Barcelona Schroth Physical Therapy School (BSPTS) and various Schroth variants share core principles of three-dimensional correction, rotational breathing, and postural awareness, which formed the basis for inclusion in this review. However, the heterogeneity observed in implementation details suggests that even within Schroth methodology, standardization remains an area requiring further development.

## 5 Conclusions

This systematic review establishes evidence-based dose-response relationships for Schroth exercise prescription in adolescent idiopathic scoliosis management. Exploratory subgroup and network meta-analyses suggest that moderate frequency interventions (3–4 sessions/week) combined with medium duration sessions (46–75 min) may represent optimal parameters for Cobb angle improvement within Schroth methodology, though these findings require confirmation through prospective trials with pre-specified dose-stratification. The identification of non-linear dose-response patterns with diminishing returns at higher frequencies challenges conventional assumptions about exercise prescription intensity and provides foundation for evidence-based clinical guidelines. The findings support sustainable exercise prescription approaches that optimize patient outcomes while minimizing treatment burden, addressing critical gaps in current clinical practice guidelines. Future research should focus on long-term effectiveness, individual response predictors, and standardized protocol development to further refine optimal Schroth exercise prescription for this prevalent adolescent condition. These recommendations apply specifically to Schroth-based interventions. Clinicians employing other PSSE methodologies should refer to method-specific evidence. The prescription protocol resulting from this analysis represents optimal parameters within Schroth methodology and cannot represent all physiotherapy approaches for scoliosis. These recommendations, while evidence-based, require validation through prospective implementation studies examining long-term effectiveness, cost-effectiveness, and patient adherence across diverse healthcare contexts, economic settings, and cultural environments. International applicability will vary based on healthcare system capacity, insurance coverage models, and physiotherapy resource availability.

## Data Availability

The original contributions presented in the study are included in the article/Supplementary Material, further inquiries can be directed to the corresponding author.

## Appendix

### Appendix 1: Search strategies for each database

**Table:**

Database	Search strategy	Results
Scopus	(TITLE-ABS-KEY ((“adolescent idiopathic scoliosis” OR “AIS” OR “juvenile idiopathic scoliosis” OR “idiopathic scoliosis” OR “adolescent scoliosis” OR “spinal curvature” OR “spinal deformity” OR “scoliosis”))) AND (TITLE-ABS-KEY ((“Schroth” OR “Schroth exercise*” OR “Schroth method*” OR “Schroth therap*” OR “Schroth technique*” OR “Schroth training” OR “physiotherapeutic scoliosis-specific exercise*” OR “physiotherapeutic scoliosis specific exercise*” OR “PSSE” OR “scoliosis-specific exercise*” OR “scoliosis specific exercise*” OR “three-dimensional exercise*” OR “3D exercise*” OR “postural correction exercise*”))) AND (TITLE-ABS-KEY ((“randomized controlled trial*” OR “randomised controlled trial*” OR “randomized” OR “randomised” OR “controlled trial*” OR “clinical trial*” OR “RCT” OR “controlled study” OR “controlled studies” OR “random allocation” OR “randomly allocated” OR “randomly assigned” OR “random assignment”))) AND PUBYEAR > 2013 AND PUBYEAR < 2026 AND LANGUAGE (english) AND (DOCTYPE (ar) OR DOCTYPE (re)) AND SRCTYPE (j) AND (LIMIT-TO (EXACTKEYWORD, “Randomized Controlled Trial”) OR LIMIT-TO (EXACTKEYWORD, “Article”) OR LIMIT-TO (EXACTKEYWORD, “Adolescent Idiopathic Scoliosis”) OR LIMIT-TO (EXACTKEYWORD, “Controlled Study”) OR LIMIT-TO (EXACTKEYWORD, “Idiopathic Scoliosis”) OR LIMIT-TO (EXACTKEYWORD, “Schroth”) OR LIMIT-TO (EXACTKEYWORD, “Schroth Exercise”) OR LIMIT-TO (EXACTKEYWORD, “Schroth Method”)) AND (LIMIT-TO (DOCTYPE, “ar”)) AND (LIMIT-TO (LANGUAGE, “English”)) AND (LIMIT-TO (SRCTYPE, “j”)) AND (LIMIT-TO (PUBSTAGE, “final”))	78
Web of science	TS = (((“adolescent idiopathic scoliosis” OR “AIS” OR “juvenile idiopathic scoliosis” OR “idiopathic scoliosis” OR “adolescent scoliosis” OR “spinal curvature” OR “spinal deformity” OR “spinal deformities” OR “scoliosis”) AND (“Schroth” OR “Schroth exercise*” OR “Schroth method*” OR “Schroth therap*” OR “Schroth technique*” OR “Schroth training” OR “Schroth approach” OR “physiotherapeutic scoliosis-specific exercise*” OR “physiotherapeutic scoliosis specific exercise*” OR “PSSE” OR “scoliosis-specific exercise*” OR “scoliosis specific exercise*” OR “three-dimensional exercise*” OR “3D exercise*” OR “postural correction exercise*” OR “corrective exercise*”) AND (“randomized controlled trial*” OR “randomised controlled trial*” OR “randomized” OR “randomised” OR “controlled trial*” OR “clinical trial*” OR “RCT” OR “controlled study” OR “controlled studies” OR “comparative study” OR “comparative studies” OR “intervention study” OR “intervention studies” OR “experimental study” OR “experimental studies” OR “random allocation” OR “randomly allocated” OR “randomly assigned” OR “random assignment” OR “double blind*” OR “single blind*” OR “placebo controlled”))) Filter: Timespan: 2014-01-01 to 2025-06-30; Document Types: Article or Clinical Trial; Languages: English	36
PubMed	(((“Scoliosis”[Mesh] OR “Spinal Curvatures”[Mesh]) OR (“adolescent idiopathic scoliosis”[Title/Abstract] OR “AIS”[Title/Abstract] OR “juvenile idiopathic scoliosis”[Title/Abstract] OR “idiopathic scoliosis”[Title/Abstract] OR “adolescent scoliosis”[Title/Abstract] OR “spinal curvature”[Title/Abstract] OR “spinal deformity”[Title/Abstract] OR “scoliosis”[Title/Abstract])) AND ((“Exercise Therapy”[Mesh] OR “Physical Therapy Modalities”[Mesh]) AND (“Schroth”[Title/Abstract] OR “Schroth exercise”[Title/Abstract] OR “Schroth method”[Title/Abstract] OR “Schroth therapy”[Title/Abstract] OR “Schroth technique”[Title/Abstract] OR “Schroth training”[Title/Abstract] OR “physiotherapeutic scoliosis-specific exercise”[Title/Abstract] OR “physiotherapeutic scoliosis specific exercise”[Title/Abstract] OR “PSSE”[Title/Abstract] OR “scoliosis-specific exercise”[Title/Abstract] OR “scoliosis specific exercise”[Title/Abstract] OR “three-dimensional exercise”[Title/Abstract] OR “3D exercise”[Title/Abstract] OR “postural correction exercise”[Title/Abstract])) AND ((“Randomized Controlled Trial”[Publication Type] OR “Controlled Clinical Trial”[Publication Type] OR “Clinical Trial”[Publication Type] OR “Random Allocation”[Mesh] OR “Double-Blind Method”[Mesh] OR “Single-Blind Method”[Mesh]) OR (“randomized controlled trial”[Title/Abstract] OR “randomised controlled trial”[Title/Abstract] OR “randomized”[Title/Abstract] OR “randomised”[Title/Abstract] OR “controlled trial”[Title/Abstract] OR “clinical trial”[Title/Abstract] OR “RCT”[Title/Abstract] OR “controlled study”[Title/Abstract] OR “random allocation”[Title/Abstract] OR “randomly allocated”[Title/Abstract] OR “randomly assigned”[Title/Abstract]))) AND (“2014/01/01”[Date - Publication]: “2025/06/30”[Date - Publication]) AND English[Language] AND (“Journal Article”[Publication Type] OR “Randomized Controlled Trial”[Publication Type] OR “Controlled Clinical Trial”[Publication Type]) NOT (“Review”[Publication Type] OR “Case Reports”[Publication Type] OR “Letter”[Publication Type] OR “Editorial”[Publication Type] OR “Comment”[Publication Type] OR “News”[Publication Type]) Filters: Clinical Trial, Randomized Controlled Trial, English, Child: birth-18 years, Exclude preprints, MEDLINE	24
SportDiscus	XB (“adolescent idiopathic scoliosis” OR “AIS” OR “juvenile idiopathic scoliosis” OR “idiopathic scoliosis” OR “adolescent scoliosis” OR “spinal curvature” OR “spinal deformity” OR “spinal deformities” OR “scoliosis) AND XB (“Schroth” OR “Schroth exercise*” OR “Schroth method*” OR Schroth therap*” OR “Schroth technique*” OR “Schroth training” OR “Schroth approach” OR “physiotherapeutic scoliosis-specific exercise*” OR “physiotherapeutic scoliosis specific exercise*” OR “PSSE” OR “scoliosis-specific exercise*” OR “scoliosis specific exercise*” OR “three-dimensional exercise*” OR “3D exercise*” OR “postural correction exercise*”) AND XB (“randomized controlled trial*” OR “randomised controlled trial*” OR “randomized” OR “randomised” OR “controlled trial*” OR “clinical trial*” OR “RCT” OR “controlled study” OR “controlled studies” OR “comparative study” OR “comparative studies” OR “intervention study” OR “intervention studies” OR “experimental study” OR “experimental studies” OR “random allocation” OR “randomly allocated” OR “randomly assigned” OR “random assignment”) Filters: Past 10 years; English; Academic Journal; Article; Clinical Trial; Journal Article; Databases SPORTDiscus with Full Text	5
